# Asterless Licenses Daughter Centrioles to Duplicate for the First Time in *Drosophila* Embryos

**DOI:** 10.1016/j.cub.2014.04.023

**Published:** 2014-06-02

**Authors:** Zsofia A. Novak, Paul T. Conduit, Alan Wainman, Jordan W. Raff

**Affiliations:** 1 Sir William Dunn School of Pathology, University of Oxford, South Parks Road, Oxford OX1 3RE, UK; 2 Oxford Micron Advanced Bioimaging Unit, Department of Biochemistry, University of Oxford, South Parks Road, Oxford OX1 3QU, UK

## Abstract

Centrioles form centrosomes and cilia, and defects in any of these three organelles are associated with human disease [1]. Centrioles duplicate once per cell cycle, when a mother centriole assembles an adjacent daughter during S phase. Daughter centrioles cannot support the assembly of another daughter until they mature into mothers during the next cell cycle [2–5]. The molecular nature of this daughter-to-mother transition remains mysterious. Pioneering studies in *C. elegans* identified a set of core proteins essential for centriole duplication [6–12], and a similar set have now been identified in other species [10, 13–18]. The protein kinase ZYG-1/Sak/Plk4 recruits the inner centriole cartwheel components SAS-6 and SAS-5/Ana2/STIL, which then recruit SAS-4/CPAP, which in turn helps assemble the outer centriole microtubules [19, 20]. In flies and humans, the Asterless/Cep152 protein interacts with Sak/Plk4 and Sas-4/CPAP and is required for centriole duplication, although its precise role in the assembly pathway is unclear [21–24]. Here, we show that Asl is not incorporated into daughter centrioles as they assemble during S phase but is only incorporated once mother and daughter separate at the end of mitosis. The initial incorporation of Asterless (Asl) is irreversible, requires DSas-4, and, crucially, is essential for daughter centrioles to mature into mothers that can support centriole duplication. We therefore propose a “dual-licensing” model of centriole duplication, in which Asl incorporation provides a permanent primary license to allow new centrioles to duplicate for the first time, while centriole disengagement provides a reduplication license to allow mother centrioles to duplicate again.

## Highlights

• Daughter centrioles do not incorporate Asterless during their assembly
• New centrioles recruit Asl once they disengage from their mother centriole
• The initial incorporation of Asl is irreversible and requires DSas-4
• Asl incorporation is essential for disengaged new centrioles to duplicate

## Graphical Abstract

**Figure:**
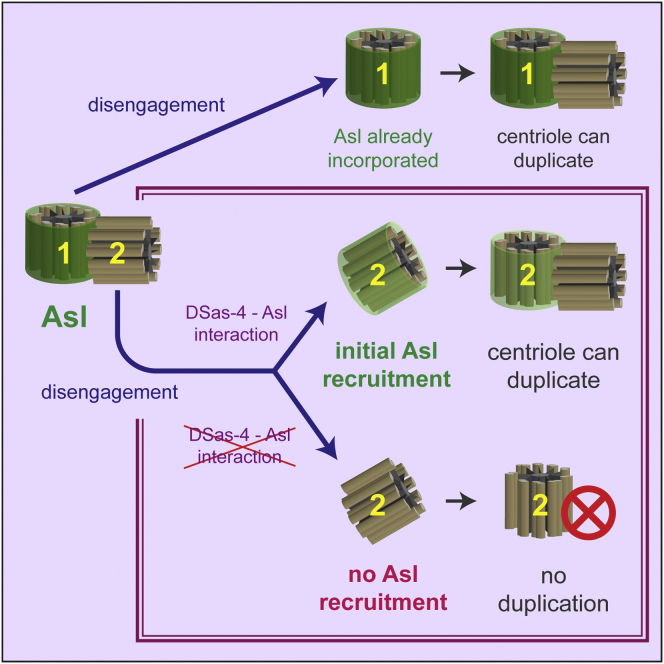

## Teaser

Novak et al. show that the key *Drosophila* centriole duplication protein Asterless is not incorporated into new centrioles as they assemble but is incorporated later once the centrioles disengage. Asl incorporation depends on DSas-4 and is essential for new centrioles to duplicate, suggesting a “dual-licensing” model of centriole duplication.

## Results

### Daughter Centrioles Incorporate DSas-4, but Not Asl, during Their Assembly

To better understand how Asl and DSas-4 might function together in fly centriole duplication, we followed the behavior of GFP-fusions of these proteins in centrosomes during the rapid, early, mitotic cycles in living syncytial blastoderm *Drosophila* embryos. For all experiments, we expressed near-endogenous levels of either DSas-4-GFP or Asl-GFP [21] in the absence of the corresponding endogenous protein (Figures S1A and S1B available online).

**Figure 1:**
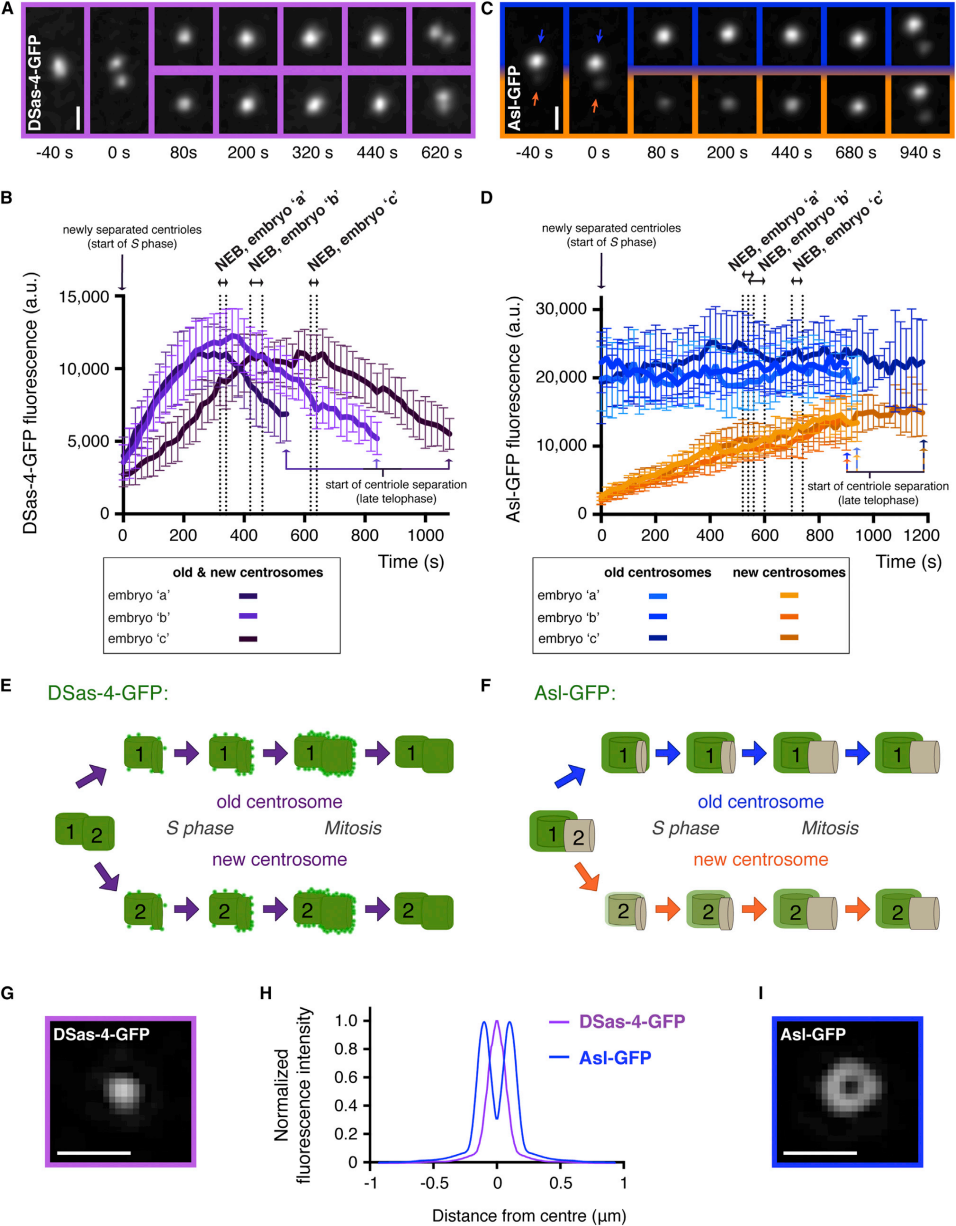
Daughter Centrioles Incorporate DSas-4, but Not Asl, during Their Assembly (A) Fluorescence images from a time-lapse movie show DSas-4-GFP incorporation into newly separated centrosomes over a single cell cycle; time (s) relative to centriole separation at t = 0 s is indicated. Scale bar, 1 μm. (B) Graph shows averaged centrosomal DSas-4-GFP fluorescence (a.u. = arbitrary units) over time from three embryos (>25 centrosomes analyzed in each). Error bars indicate the SD. (C and D) Images (C) and graph (D) show Asl-GFP incorporation into newly separated centrosomes, presented as in (A) and (B), respectively. Note that new centrosomes (orange box and graph) have not reached as high a level of fluorescence as the old centrosomes (blue box and graph) by the end of the cycle; this is because new centrosomes continue to incorporate some Asl-GFP in the following cell cycle (Figure S2E). For this reason, the analysis of old centrosomes shown here focuses on older (i.e., brighter) centrosomes that had already reached their full brightness (see Figure S2E legend for more detail). (E and F) Schematic interpretation of how DSas-4-GFP (E) and Asl-GFP (F) incorporate into centrioles. (G–I) Three-dimensional structured illumination microscopy (3D-SIM) superresolution images of centriolar DSas-4-GFP (G) or Asl-GFP (I) in living embryos (see the Supplemental Experimental Procedures for a full explanation of how these data were obtained and analyzed). Scale bars, 0.5 μm. (H) Graph shows the average centriolar fluorescence intensity profiles of DSas-4-GFP (purple, n = 8) and Asl-GFP (blue, n = 24) in 3D-SIM images. Note that in (E), the immobile fraction of DSas-4-GFP is shown associated with the centrioles, while, for simplicity, the mobile fraction (that subsequently dissociates during mitosis) is shown tightly surrounding the centrioles based on our localization data (G and H). See also Figures S1 and S2 and the Supplemental Experimental Procedures.

In early S phase, just after the centrosomes have separated (Figure 1A, t = 0 s), the level of DSas-4-GFP fluorescence was similar at the two centrosomes and gradually increased during S phase, as new daughter centrioles assembled (Figures 1A and 1B). DSas-4-GFP levels plateaued shortly before the start of mitosis (nuclear envelope breakdown [NEB]; Figures 1A and 1B), when new daughter centrioles have reached their full size [25]; the fluorescence then steadily declined as mitosis proceeded. This behavior suggests that a pool of DSas-4 is stably incorporated into daughter centrioles as they form but that some “excess” DSas-4 is recruited during S phase and then lost during mitosis (Figure 1E). Fluorescence recovery after photobleaching (FRAP) experiments strongly supported this interpretation (Figures S1C–S1G).

Previous superresolution microscopy studies have shown that DSas-4 is tightly concentrated at centrioles in fly cells and does not spread into the pericentriolar paterial (PCM); it localizes within an outer ring of Asl that lies at the outer edge of the centrioles [26, 27]. We confirmed that this was also the case for the DSas-4-GFP and Asl-GFP fusion proteins in living embryos using 3D-structured illumination superresolution microscopy (Figures 1G–1I). Note that our superresolution images of DSas-4-GFP and Asl-GFP reveal the localization of the C terminus of both proteins, which are not predicted to colocalize: the C terminus of DSas-4 interacts with the N terminus of the centriole cartwheel protein Ana2 [28] and so would be predicted to lie internally to the C terminus of Asl, which is what we observe. Thus, we are confident that DSas-4-GFP is a bona fide marker of centrioles in these embryos.

In contrast to DSas-4, we observed dramatically different levels of Asl-GFP at the two separating centrioles in early S phase (Figure 1C, t = 0 s). An analysis with the centriole-age marker RFP-PACT [29] revealed that the centrosome that inherited the original mother centriole (hereafter the “old” centrosome) always exhibited more Asl-GFP than the centrosome that inherited the original daughter centriole (hereafter the “new” centrosome) (Figures S2A and S2B). Asl-GFP fluorescence in new centrosomes (Figures 1C and 1D, orange labels) steadily increased throughout S phase and into mitosis. Surprisingly, Asl-GFP fluorescence in the oldest centrosomes (Figures 1C and 1D, blue labels) did not appear to increase at all, even though these old centrosomes formed new daughters during this time. This strongly suggests that new daughter centrioles do not incorporate Asl-GFP while they assemble and that the incorporation of Asl-GFP we observe at new centrosomes (orange labels, Figures 1C and 1D) must be due to incorporation at the new mother centrioles (Figure 1F). We conclude that although Asl is essential for centriole duplication [21], Asl-GFP is surprisingly not incorporated into daughter centrioles as they assemble during S phase but only starts to be incorporated at about the time they separate from their mothers at the end of mitosis.

FRAP analysis of Asl-GFP at old centrosomes (where total GFP fluorescence levels are constant; Figures 1C and 1D) revealed that Asl-GFP fluorescence recovered after bleaching but plateaued at ∼50% recovery (Figures S2C and S2D). Thus, there are two fractions of Asl-GFP at mother centrosomes: ∼50% appears to be irreversibly incorporated (hereafter the “immobile” fraction), while ∼50% can still exchange with the cytosolic pool (hereafter the “mobile” fraction). Similar results were obtained for new centrosomes, but this analysis was complicated because new centrosomes also incorporate additional Asl-GFP protein during the cell cycle (Figures 1C and 1D and data not shown).

### The Initial Incorporation of Asl into Newly Formed Centrioles Depends on DSas-4

The C-terminal region of Asl can interact with the N-terminal region of DSas-4 [22], and superresolution microscopy has revealed that these interacting domains precisely colocalize at the centriole wall [26]. We therefore tested whether DSas-4 might be required to recruit Asl to new centrioles at the end of mitosis.

**Figure 2:**
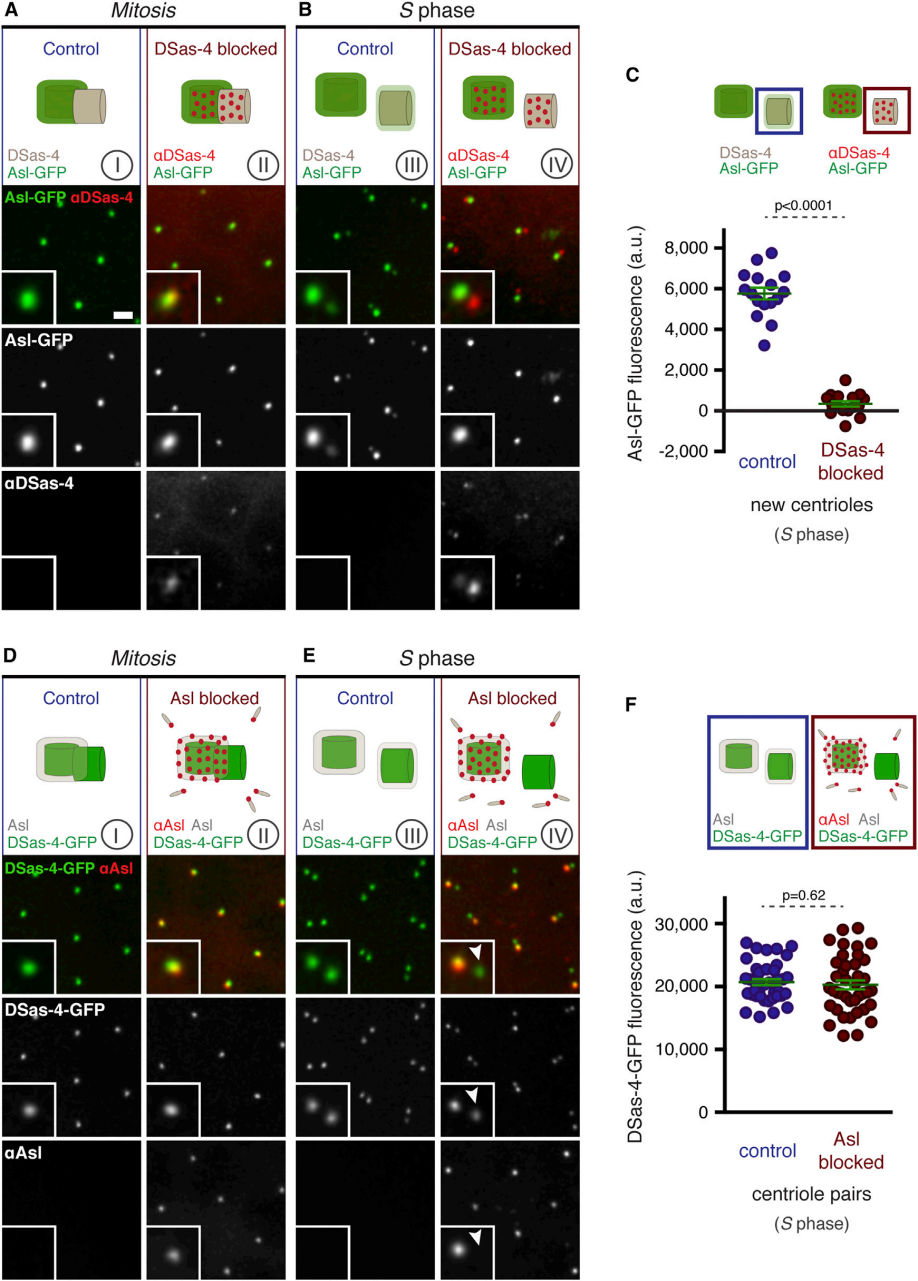
The Initial Incorporation of Asl-GFP into Newly Assembled Centrioles Can Be Inhibited with Anti-DSas-4 or Anti-Asl Antibodies (A and B) Fluorescence images show two regions (“control” and “DSas-4-blocked”) of an embryo expressing Asl-GFP (green) that has been injected with Texas-red-labeled anti-DSas-4 antibodies (red) at the start of mitosis (columns I and II) and several minutes later after the centrioles have separated at the start of S phase (columns III and IV). The control region (columns I and III) is far from the injection site (see Figures S3B and S3C), so no antibodies are detectable; the DSas-4-blocked region (columns II and IV) is close to the injection site, and the antibodies bind to the centrioles. The schematic at the top of each panel illustrates how the DSas-4 antibodies bind to centrioles in mitosis (II) and block the subsequent incorporation of Asl-GFP into the new centriole at the start of S phase, but they do not interfere with Asl-GFP localization at the old centriole (IV). (C) The graph quantifies Asl-GFP levels in new centrioles in early S phase in the control region and in the DSas-4-blocked region (n = 16 centrioles from three injected embryos), as shown schematically above the graph. Error bars indicate the SEM. (D and E) Fluorescence images show two regions of an embryo expressing DSas-4-GFP (green) injected with Texas-red-labeled anti-Asl antibodies (red), presented as in (A) and (B) above. The schematics illustrate how the anti-Asl antibodies do not perturb the localization of DSas-4-GFP that is already incorporated into the centrioles at the time of antibody injection but bind the endogenous (nonfluorescent) Asl molecules (gray) in the cytoplasm and so block their incorporation into the new centriole. (F) The graph quantifies DSas-4-GFP levels in centriole pairs in early S phase in either control or Asl-blocked regions (n = 40 centriole pairs from four embryos), as illustrated schematically above the graph. Note that we compare centriole pairs instead of individual centrioles in this experiment because we cannot distinguish old and young control centrioles based on DSas-4-GFP levels alone. Scale bars, 2 μm. Error bars indicate the SEM. See also Figure S3 and the Supplemental Experimental Procedures.

We injected Texas-red-labeled, affinity-purified antibodies raised against the N-terminal region of DSas-4 (that should interfere with the binding of DSas-4 to Asl; Figure S3A) into embryos expressing Asl-GFP. This was done just as the embryos entered mitosis, when daughter centrioles had fully formed [25] (and so had incorporated DSas-4) but had not yet separated from their mothers (and so had not yet incorporated Asl). The antibodies bound to the centrioles that were close to the injection site (Figure 2A, DSas-4 blocked; Figure S3B), but not to centrioles that were further away from the injection site (Figure 2A, “control”; Figure S3B). By the time the centrioles had separated at the start of S phase (Figures 2B and S3C), Asl-GFP localized to both old and new centrosomes in the control region but failed to localize to the new centrosomes in the DSas-4-antibody-blocked region (Figures 2B and 2C); importantly, new centrosomes were decorated with anti-DSas-4 antibodies, indicating that new centrosomes were present and the new mother centrioles in these centrosomes had successfully incorporated DSas-4. We conclude that DSas-4 is incorporated into new centrioles before Asl and that the anti-DSas-4 antibodies block the interaction between DSas-4 and Asl, and so block the subsequent incorporation of Asl into new centrioles at the end of mitosis (see schematic illustration, Figure 2B). We cannot formally exclude the unlikely possibility, however, that the anti-DSas-4 antibodies block the incorporation of Asl into new centrioles by a mechanism that does not depend on their blocking the interaction of DSas-4 with Asl.

We noticed that the anti-DSas-4 antibodies did not detectably perturb the localization of Asl-GFP in old centrosomes, even though their centrioles were decorated with the antibodies (Figure 2B). FRAP experiments indicated that the mobile fraction of Asl-GFP in old centrosomes continued to turn over with near-normal kinetics, despite the presence of the antibodies (Figure S3D). Thus, although anti-DSas-4 antibodies successfully block the initial recruitment of Asl to new centrioles, they do not block either the recruitment of the mobile Asl fraction to old centrioles or the maintenance of the immobile Asl fraction there.

### Asl Incorporation Allows Newly Assembled, Disengaged Centrioles To Duplicate for the First Time

Asl is essential for centriole duplication in flies [21, 30], so we reasoned that its incorporation into newly assembled centrioles might be required to allow them to mature into mothers competent for duplication in the next cell cycle. To investigate this possibility, we needed to specifically block the incorporation of Asl into newly disengaged daughter centrioles. As just described, injections of anti-DSas-4 antibodies did block this incorporation (Figures 2A–2C), and we showed previously that these antibodies also block centriole duplication [17]. Because DSas-4 itself is essential for centriole duplication, however, we could not be sure that it was the lack of Asl incorporation into new centrioles that was blocking their subsequent duplication. We needed to block Asl incorporation without directly interfering with DSas-4 function.

We therefore used Texas-red-labeled, affinity-purified antibodies raised against the C-terminal region of Asl, which should interfere with its binding to DSas-4 (Figure S3A). We injected the antibodies into DSas-4-GFP-expressing embryos that were entering mitosis. The antibodies bound to centrosomes close to the injection site (Figure 2D), and, as the centrosomes separated at the end of mitosis, DSas-4-GFP fluorescence was localized to both old and new centrosomes, indicating that the antibodies did not interfere with the localization of DSas-4-GFP molecules that had already been incorporated into the centrioles at the time of the antibody injection (Figures 2E and 2F). We noticed, however, that the anti-Asl antibodies often decorated only one centrosome of the separating pair (Figure 2E)—presumably the old centrosome, because it contains the original mother centriole that would have already incorporated Asl at the time of the antibody injection. This finding suggests that the antibodies bind to Asl molecules in the mother centrioles but also to Asl molecules in the cytoplasm, thereby blocking their incorporation into the newly assembled centrioles (see schematic illustration, Figure 2E).

**Figure 3:**
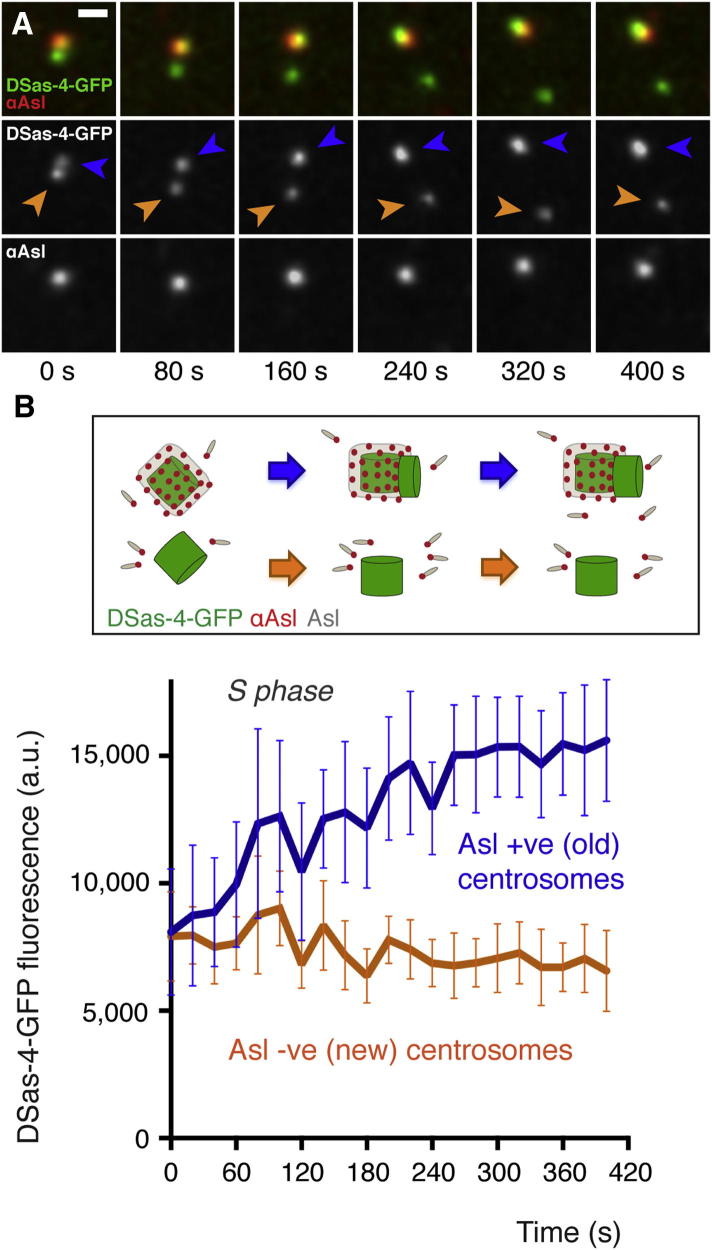
Asl Recruitment to New Centrioles Is Required for Their Duplication (A) Fluorescence images from a time-lapse movie of an embryo expressing DSas-4-GFP (green) that has been injected with Texas-red-labeled anti-Asl antibodies (red); time (s) relative to centriole separation at t = 0 s is indicated. DSas-4-GFP is incorporated into newly separated old centrosomes (blue arrowheads), even though these centrioles are strongly decorated with anti-Asl antibodies. DSas-4-GFP is not, however, incorporated into newly separated young centrosomes (orange arrowheads), even though no anti-Asl antibodies are detectably binding to these centrosomes. Scale bar, 1 μm. (B) Graph (with explanatory schematic) quantifies the incorporation of DSas-4-GFP into old anti-Asl-decorated centrosomes (blue line); there is no incorporation into young Asl-negative centrosomes (orange line). Error bars indicate the SEM. See also Figure S3 and the Supplemental Experimental Procedures.

To test whether these newly separated centrioles that lacked Asl could support centriole duplication, we compared the incorporation of the centriole marker DSas-4-GFP at old centrosomes (that have inherited an old mother centriole containing both DSas-4 and Asl) and at new centrosomes (that have inherited a new mother centriole containing DSas-4, but not Asl [arrowhead, Figure 2E]). Despite being heavily decorated with anti-Asl antibodies, old centrosomes incorporated additional DSas-4-GFP, indicating that mother centrioles were assembling new daughters (Figures 3 and S3F, blue labels). In contrast, even though new centrosomes were not detectably decorated with anti-Asl antibodies, they did not incorporate any additional DSas-4-GFP, indicating that these new mother centrioles could not assemble daughters (Figures 3 and S3F, orange labels). Thus, new centrioles that lack Asl appear unable to initiate centriole duplication, even though they have disengaged from their mothers and have passed through mitosis.

## Discussion

**Figure 4:**
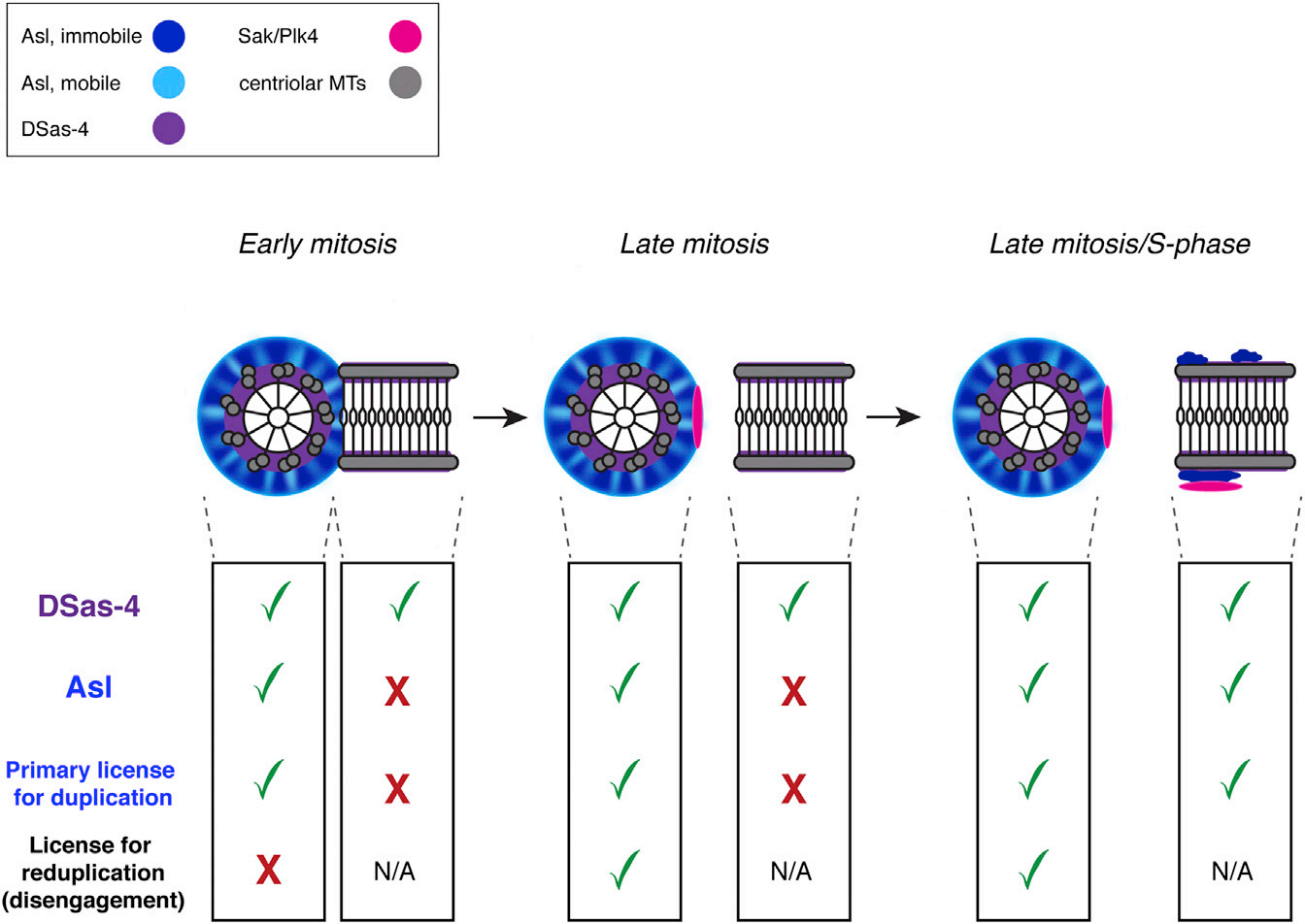
A Dual Licensing Model of Centriole Duplication The schematic diagrams illustrate a centriole pair, with mother in end-on view (left) and daughter in side-on view (right), as they pass through mitosis. The table below illustrates the state of each centriole in terms of DSas-4 incorporation, Asl incorporation, and whether the centriole has a primary license for its first duplication or a reduplication license for subsequent rounds of duplication. In early mitosis, the mother centriole has incorporated immobile Asl during a previous cell cycle, which irreversibly provided it with a primary license, but it is unable to duplicate again because it lacks a reduplication license, which it will acquire when it disengages from its daughter [4, 5, 37]. The daughter centriole has incorporated DSas-4, but not Asl, and so it lacks a primary license and cannot duplicate until it disengages and matures into a mother. In late mitosis, the centrioles disengage: the mother centriole thereby acquires a reduplication license (speculatively illustrated here by a free “patch” of Sak on its side); the separated daughter centriole cannot duplicate until it starts to incorporate Asl (shown here occurring in late mitosis/S phase), which allows it to recruit Sak for the first time. In *Drosophila* blastoderm embryos, Asl incorporation starts around the time centrioles disengage at the end of mitosis; because centriole disengagement is also closely linked to S phase initiation, we cannot tell whether Asl incorporation is triggered by centriole disengagement or by S phase initiation.

Our observations demonstrate that Asl recruitment to disengaged new centrioles has a critical role in allowing these centrioles to mature into mothers that can duplicate for the first time. During all subsequent duplication cycles, however, mother centrioles already contain a pool of immobile Asl, and this appears to be sufficient to allow subsequent rounds of duplication, because anti-Asl antibodies block the recruitment of the mobile fraction of Asl to mother centrioles (Figure S3E) but do not block their duplication (Figure 3). For an old centriole to duplicate again, therefore, disengagement of the daughter centriole appears to be the crucial licensing event that allows reduplication [4, 5, 31], because immobile Asl incorporation has already occurred. Taken together, our findings suggest a dual-licensing model in which the recruitment of the immobile fraction of Asl by DSas-4 provides an irreversible primary license to allow newly formed centrioles to duplicate for the first time, while centriole disengagement provides a reduplication license [5] to allow older centrioles to duplicate again (Figure 4).

How might Asl perform this primary licensing function? In flies, Asl localizes Sak to centrioles [22], probably explaining why Asl incorporation is a crucial step in converting a disengaged daughter centriole into a mother centriole that can duplicate. Cep152 (human Asl) is also required for the efficient loading of Plk4 (human Sak) onto centrioles in vertebrate cells [22–24], although it appears to share this function with Cep192 (human SPD-2) [32, 33]. Our model is consistent with superresolution microscopy studies on fixed cells, which show that Asl/Cep152 is associated with the mother centriole in an engaged centriole pair [27, 32, 34, 35], suggesting that a similar model may operate in vertebrates. Although the primary and reduplication licensing steps are mechanistically different, we suspect that they share a common purpose: to provide an Asl platform that is competent to recruit Sak to initiate daughter centriole assembly (Figure 4).

Our model can explain why only mother centrioles can support certain types of experimentally induced centriole reduplication, including that induced by Sak overexpression [2, 3] or by ablation of one of the engaged centrioles during an arrested S phase [4]. It can also explain why daughter centrioles appear to have to be “modified” before they can support any duplication [5]; our results strongly suggest that this modification, at least in flies, is Asl incorporation.

How is Asl recruited to centrioles? We speculate that DSas-4 initially recruits the immobile fraction of Asl, which then recruits the mobile fraction. This would explain the 50:50 ratio of immobile to mobile Asl (Figures S2C, S2D, S3D, and S3E). Our finding that anti-Asl antibodies strongly block the recruitment of the mobile fraction of Asl to mother centrosomes (Figure S3E) also supports this possibility. It is tempting to speculate that the mobile fraction of Asl may be important for the previously described role of Asl in mitotic PCM recruitment [22, 29, 36]. It is also interesting to note that only very low levels of Asl seem to be required at new mother centrioles to allow duplication (Figures 1C and 1D).

It remains to be determined what regulates the interaction between DSas-4 and Asl such that Asl is only recruited to daughter centrioles at about the time they separate from their mothers. We speculate that the phosphorylation state of either or both proteins could be altered at the end of mitosis, perhaps increasing the affinity of their interaction. Polo/Plk1 seems to play a crucial part in resetting the reduplication license at old centrioles through the regulation of centriole disengagement [37]; perhaps it also has an important role in the primary licensing of new centrioles by regulating the interaction between DSas-4 and Asl.

## Supplemental Information

Document S1. Figures S1–S3 and Supplemental Experimental Procedures

Document S2. Article plus Supplemental Information
