# Supplementary material for: Asterless Licenses Daughter Centrioles to Duplicate for the First Time in Drosophila Embryos
Source: Curr Biol. 2014 Jun 2;24(11):1276–82. doi: 10.1016/j.cub.2014.04.023 (PMC4046630; doi:10.1016/j.cub.2014.04.023)
Supplement: Document S1. Figures S1–S3 and Supplemental Experimental Procedures [file mmc1.pdf]

Current Biology, Volume *24*

Supplemental Information

Asterless Licenses Daughter Centrioles to Duplicate for the First Time in *Drosophila* Embryos

Zsofia A. Novak, Paul T. Conduit, Alan Wainman, and Jordan W. Raff

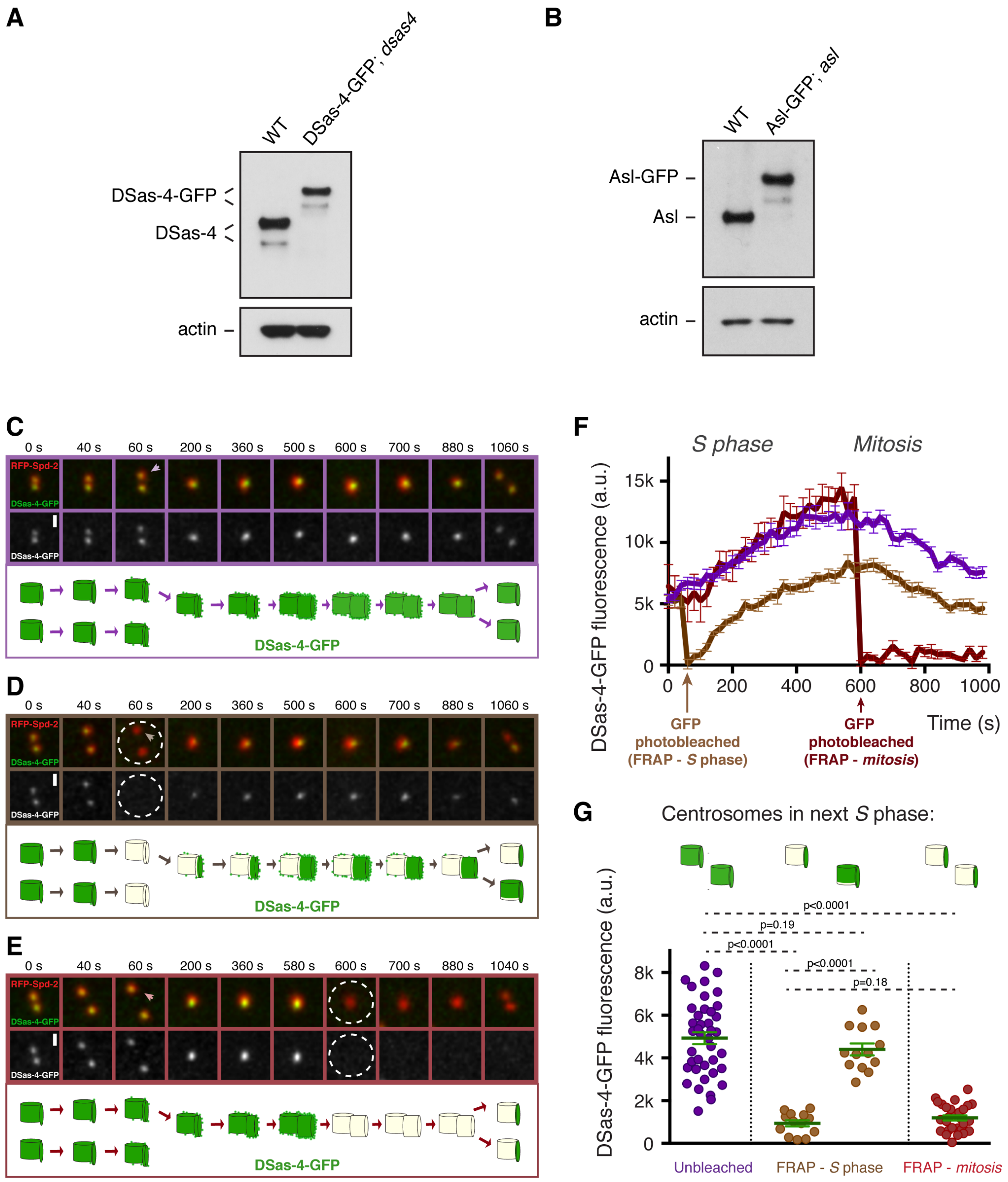

Figure S1. DSas-4-GFP is stably incorporated into growing centrioles during *S*-phase and excess DSas-4-GFP is lost from centrioles during mitosis (related to Figure 1). (A,B) Western blot analyses of embryo extracts shows that, in the embryos used for our analyses, DSas-4-GFP (A) and Asl-GFP (B) are expressed at similar levels to the corresponding endogenous protein in wild-type embryos. Representative blots from 3 independent repeats of each experiment are shown. (C-E) Fluorescence images from time-lapse FRAP movies show DSas-4-GFP (*green* in merged panels)

incorporation into newly separated (t=0s) control (non-bleached) centrosomes (C), centrosomes that were bleached in early *S*-phase (t=60s) (D), or centrosomes that were bleached in mitosis (t=600s) (E). The arrows at t=60s identify the centrosome from the separating pair that was followed in the subsequent panels. These embryos also expressed the centrosome marker RFP-DSpd-2 (*red*) so that centrioles could be followed even after photobleaching. A schematic interpretation of the distribution of DSas-4-GFP at each time point is shown below each set of fluorescence panels. Scale bars: 1μm. (F) Graph shows the quantification through time of DSas-4-GFP fluorescence levels at non-bleached centrosomes (*purple*, n=8 centrosomes from 2 embryos), centrosomes bleached in *S*-phase (*brown*, n=7 centrosomes from 2 embryos) and centrosomes bleached in mitosis (*red*, n=4 centrosomes from 2 embryos). The colours of the lines match the colours of the boxes surrounding the images shown in (C-E). Error bars represent s.e.m. Note how the centrosomes bleached in *S*-phase (*brown* line) show a very similar pattern of incorporation as the control centrosomes (*purple* line), although they are less bright, presumably because the DSas-4-GFP incorporated into the mother centriole does not turn over, and so its fluorescence has been irreversibly bleached (as depicted in the cartoon in (D)). This interpretation is supported by the observation that the centrosomes bleached in mitosis do not detectably incorporate DSas-4-GFP during mitosis. (G) Quantification of centrosomal DSas-4-GFP fluorescence at the start of the next *S*-phase for centrosomes that were unbleached (*purple*, n = 40 centrosomes from 4 embryos), bleached in the previous *S*-phase (*brown*, n = 26 centrosomes from 4 embryos), or bleached in the previous mitosis (red, n = 28 centrosomes from 4 embryos). Error bars represent s.e.m. Note how centrosomes bleached in the previous *S*-phase are highly asymmetric in fluorescence, presumably due to the irreversible bleaching of the mother centriole in *S*-phase.

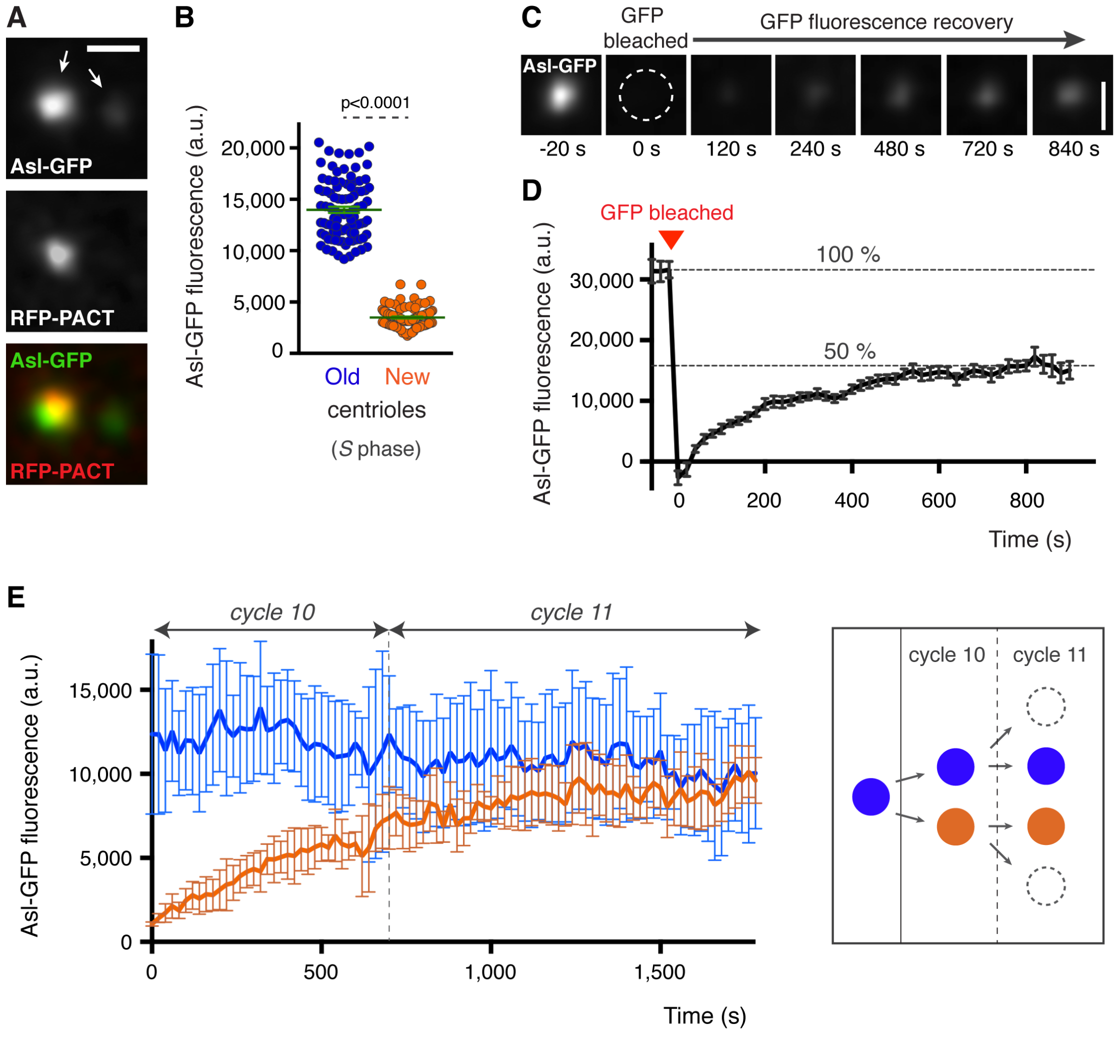

Figure S2. Further characterization of Asl-GFP behavior in living embryos (related to Figure 1).

(A) Images show the levels of Asl-GFP and RFP-PACT in two newly separated centrosomes in early *S*-phase. RFP-PACT fluorescence preferentially marks the older centriole [1], confirming that the centrosome with the older centriole contains more Asl-GFP. Scale bar: 1µm. (B) Graph quantifying Asl-GFP fluorescence levels at old and new centrioles (n=95 centriole pairs from 4 embryos). The old and new centrioles were distinguished based on RFP-PACT fluorescence. Statistical significance was calculated using a paired two-tailed t-test. Error bars represent s.e.m. (C,D) Images (C) and graph (D) show Asl-GFP fluorescence recovery following photobleaching of the Asl-GFP signal at old centrosomes at the start of *S*-phase. Time relative to photobleaching at t=0s is shown under each image. Scale bar: 1µm. Note how Asl-

GFP fluorescence recovers to only ~50% of the original pre-bleached levels; as total Asl-GFP fluorescence levels are not increasing at these centrosomes (Figure 1D, *blue* line), this indicates that ~50% of the Asl-GFP is turning over, while ~50% is immobile. In (D), the averaged fluorescence of 17 centrosomes from 3 embryos is shown. Error bars represent s.e.m. (E) Graph shows the averaged centrosomal Asl-GFP fluorescence (a.u.) over two complete nuclear cycles in a representative embryo (n=7 centrosomes each for old and new centrosomes). Error bars represent s.d. Multiple embryos were analyzed and they each showed the same qualitative result, a single embryo is shown for clarity. The graph illustrates how the old centrosomes (*blue*) maintain a relatively constant level of fluorescence over time, while the new centrosomes (*orange*), gradually accumulate fluorescence over two cycles; fluorescence levels at these new centrosomes plateau at approximately the same level as the old centrosomes towards the end of the second cycle. Because new centrosomes incorporate Asl-GFP over two cycles, if we simply compare the incorporation of Asl at *all* old and new centrosomes (in an unbiased manner), then the old centrosomes, on average, slightly increase in fluorescence due to the incorporation of Asl-GFP into the old centrosomes that are only one cycle old. For this reason, we only compare Asl-GFP levels in new centrosomes and the brightest (i.e., oldest) old centrosomes in Figure 1D (and here in Figure S2E), as these oldest centrosomes have stopped incorporating Asl-GFP. This analysis most clearly reveals that no Asl-GFP is being incorporated into the newly forming daughter centrioles, as the analysis is not complicated by the low level of incorporation into mother centrioles that are only one cell cycle old. Note that, although photobleaching is corrected for in these analyses, the centrosomes get slightly dimmer during the extended time course of these experiments (most easily visualized by the slow decline in fluorescence of the oldest centrosomes (*blue*) in (E)). This is a consistent feature that we see in extended time course experiments with many GFP-fusion proteins, and we suspect it occurs because the autofluorescence of the embryo bleaches more slowly than the GFP fluorescence; thus, our correction for photobleaching (which is based on photobleaching of the whole embryo) slightly under-corrects for the photobleaching of GFP.

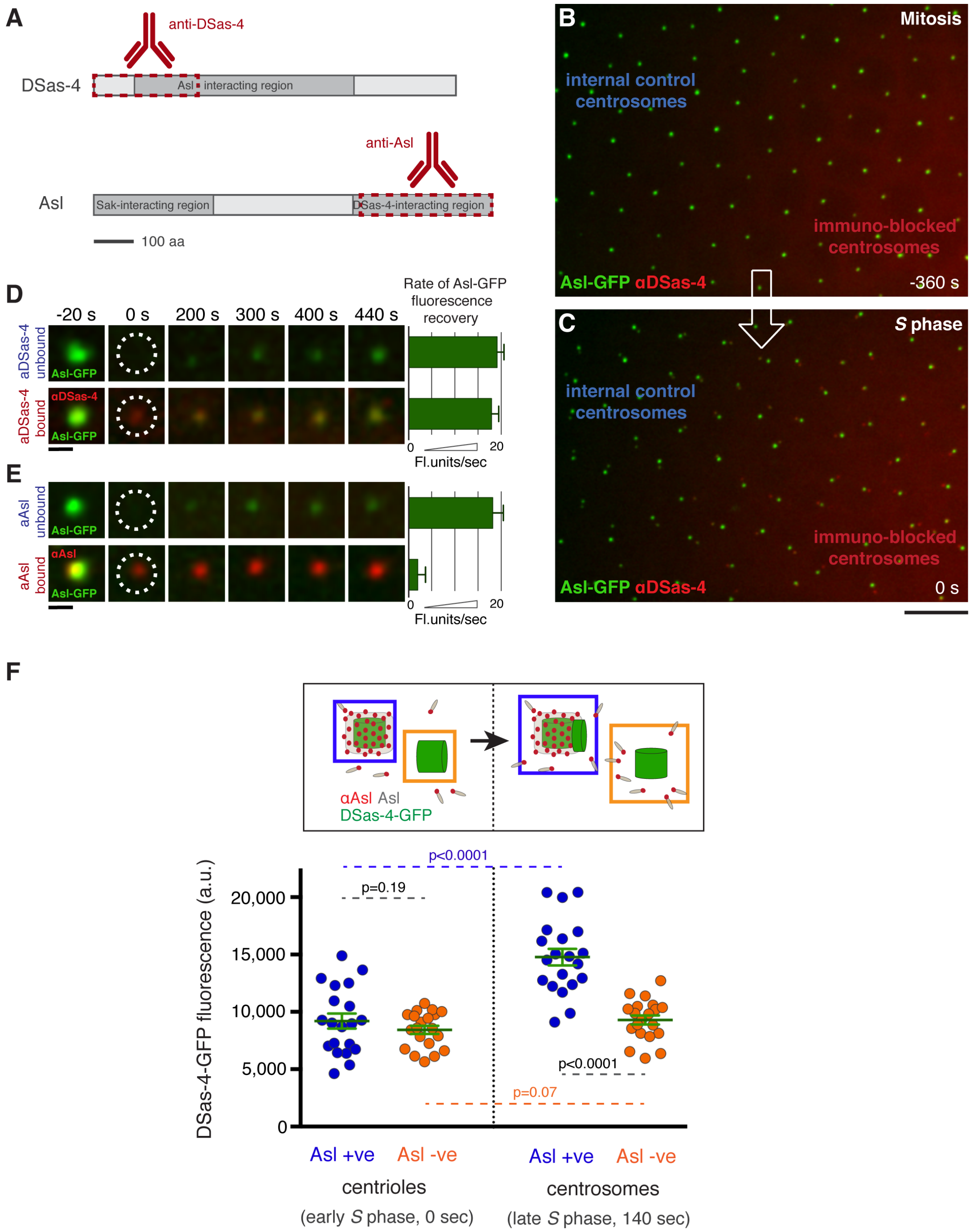

Figure S3. Multiple molecular mechanisms contribute to the recruitment of Asl to centrosomes (related to Figure 2 and Figure 3). (A) Schematic illustration of the regions of DSas-4 and Asl required for their interaction with each other and for the interaction of Asl with Sak/Plk4 [2]. Red boxes indicate the regions of DSas-4 and Asl, respectively, against which the inhibiting antibodies used in our experiments were raised. (B,C) Images show an example of an embryo antibody injection

experiment. The embryo shown here expressed Asl-GFP (*green*) and was injected during mitosis (B) with Texas-Red-labelled anti-DSas-4 antibodies (*red*) in the bottom right corner. The centrosomes close to the injection site are exposed to a high concentration of the antibodies, which binds to the centrosomes; the centrosomes further away show no detectable antibody signal and serve as internal controls. As the centrosomes separate in *S*-phase (C), Asl-GFP is detectable on the new centrosomes in the control area but not near the injection site, where the anti-DSas-4 antibodies decorate the new centrosomes—this is more easily seen in the high magnification images shown in Figure 2A. Scale bar: 10μm. (D,E) Images and graphs show Asl-GFP fluorescence (green) recovery rates following photobleaching of Asl-GFP at old centrosomes, either in a control area or an area near to the injection site following an injection of Texas-Red-labelled antibodies against either DSas-4 (D) or Asl (E); near the injection site, the old centrosomes are decorated with the injected antibodies (red). The initial recovery rates (fluorescence units/sec, during the first 180s after photobleaching) are shown in the graphs to the right of the images. The anti-DSas-4 antibodies (D) do not significantly perturb the turnover of mobile fraction of Asl (n=8 centrosomes from 3 embryos; p=0.58), while the anti-Asl antibodies (E) strongly perturb the turnover of the mobile fraction of Asl (n=8 centrosomes from 3 embryos; p=< 0.0001). Scale bars: 1μm. Error bars represent s.e.m. (F) Graph (with explanatory schematic) showing that old centrioles (that contain Asl—*blue*) and new centrioles (that lack Asl—*orange*) contain similar levels of DSas-4-GFP fluorescence at the time when they first separate and start duplication in early *S*-phase. Later in *S*-phase, the old centrosomes (*blue*) have incorporated additional DSas-4-GFP, indicating that new daughter centrioles have started to assemble, despite the presence of the anti-Asl antibodies; in contrast, the new centrioles (orange) have not incorporated any additional DSas-4, indicating that they are not assembling new daughter centrioles (n=20 centrosomes from 2 embryos for each category). Time relative to centriole separation at t=0 is shown in brackets under the graph. Statistical significance was calculated using a paired two-tailed *t*-test for each comparison. Error bars represent s.e.m.

SUPPLEMENTAL EXPERIMENTAL PROCEDURES

Fly strains and genetics. The Asl-GFP [3] and RFP-PACT [4] constructs used in this study have been described previously. The DSas-4-GFP construct was made by cloning the genetic region of *dsas-4*, from 2kb upstream of the start codon up to the stop codon, into the pUAST-GFPCT vector (Drosophila Genomics Resource Centre, pTWG-1076). The RFP-DSpd-2 construct was generated by cloning the *dspd-2* coding sequence into the pWUbq-RFP(NT) [4] Gateway vector. Transgenic lines were generated at the Department of Genetics, University of Cambridge. For all experiments, Asl-GFP was expressed in an asl^{B46} genetic null mutant background (in which the 5' UTR as well as the first 1775 bp of the *asl* coding region have been deleted — Janina Baumbach, A. W., Z.A.N. and J. W. R., manuscript in preparation), while DSas-4-GFP was expressed in a $sas4^{S2214}$ mutant [5] background.

Microscopy. 1-2h old living embryos were dechorionated by hand on sticky tape, mounted on a thin film of glue (sticky tape dissolved in heptane) on a glass bottom dish, covered with Voltalef oil and imaged immediately at 22^0C. For all imaging experiments shown in this study, with the exception of the super resolultion analysis, embryos were imaged on a Perkin Elmer ERS Spinning Disc confocal system on a Zeiss Axiovert microscope (using a 63x, 1.4NA, oil objective and a charge-coupled device camera: Orca ER, Hamamatsu Photonics). 15-17 confocal sections (0.5µm steps) were collected every 20 seconds. For photobleaching the GFP signal of individual centrosomes, a focused 488nm (when imaging GFP alone) or 440nm laser (when imaging Texas Red or RFP together with GFP) was used. For super resolution analyses (Figure 1 G-I) living embryos were imaged on OMX V3 (GE Healthcare, USA) system (60x, 1.35NA oil objective). 20-23 optical sections were collected to create each image (0.125µm steps).

Image analysis. All images acquired on the Perkin Elmer ERS Spinning Disc confocal system were analysed using Volocity software (Perkin Elmer, USA). To evaluate the changes in Asl-GFP or DSas-4-GFP fluorescence levels during the mitotic cycle, centrosomal fluorescence was calculated as a sum intensity of every centrosomal voxel for each channel (a centrosomal voxel was defined as having an intensity >4.5 standard deviations (SDs) above the mean intensity of all voxels within

the image; this setting was chosen because it selects centrosomal pixels most accurately as judged by eye while allowing consistent thresholding of centrosomal pixels above background fluorescence). Cytoplasmic background fluorescence was then subtracted from centrosomal fluorescence values (background fluorescence within centrosome at timepoint n = mean cytoplasmic fluorescence in timepoint n * number of centrosomal voxels in timepoint n). At each time point centrosomal fluorescence values were corrected for bleaching during sampling using the following method: corrected centrosomal fluorescence at timepoint n = centrosomal fluorescence at timepoint n / (total fluorescence of image at timepoint n/total fluorescence of image at timepoint 1). For DSas-4-GFP, >20 centrosomes were analysed in each of 12 embryos in total (3 embryos (>25 centrosomes each) shown in Figure 1B). For Asl-GFP, 15-25 centrosomes were analysed from each of 10 embryos in total (3 embryos (>15 centrosomes each) shown in Figure 1D).

For comparing Asl-GFP or RFP-PACT levels of centrosomes in *S*-phase (Figure S2B), centrosomal fluorescence was calculated as described above and then local cytoplasmic background fluorescence was subtracted from these values. Fluorescence values of old and young centrosome pairs were compared using a paired *t*-test.

To measure centrosomal Asl-GFP or DSas-4-GFP levels in all antibody-injection experiments (Figures 2C,F; 3B; S3F) or any FRAP experiments (Figures S1F,G; S2D; S3D,E), centrosomal fluorescence was calculated using a different method: we measured the total GFP fluorescence in a boxed region centered around the brightest centrosomal voxel and subtracted the local cytoplasmic background fluorescence. Fluorescence values of inhibited and control centrosomes (Figure 2C,F) were compared using a *t*-test (unpaired). To compare Asl-GFP fluorescence recovery in all antibody-injection experiments (Figure S3D,E) the initial rate of Asl-GFP recovery was calculated by linear regression analysis on fluorescence values measured during the first 180 seconds after photobleaching (10 measurements). A total of 8 centrosomes were analysed from 3 embryos for each group, and recovery rates were compared using a *t*-test (unpaired). To assess DSas-4-GFP recruitment to centrosomes following Asl inhibition, >25 centrosome pairs were analysed from 3 embryos in total. All 3 embryos showed the same qualitative result; in Figure 3B, data from a single embryo (10 Asl-positive and 10 Asl-negative centrosomes) is shown, as the

continuous tracking of Asl-negative centrosomes in the other embryos was more affected by the defective separation and movement of these centrosomes (most likely due to the recruitment of only minimal amounts of PCM in the absence of Asl), in Figure S3F 20 Asl-positive and 20 Asl-negative centrosomes are shown in early (0 sec) and late *S*-phase (140 sec later), from 2 embryos. Fluorescence values were compared using a paired *t*-test.

In all graphs that display data collected from individual embryos (Figure 1B,D; Figure 3B, Figure S2E) the error bars represent s.d., while in all graphs that display data pooled from multiple embryos (Figure 2C,F; Figure S1F,G; S2B,D; Figure S3D,E,F) the error bars represent s.e.m.

All super-resolution images acquired on the OMX V3 system were processed and reconstructed using SoftWorx software. For further analyses, Image J software was used for plotting the distribution of DSas-4-GFP and Asl-GFP within centrosomes. Images were processed as maximum intensity projections; each pixel of the image was sub-divided into 10x10 pixels before the radial intensity profile was calculated from the centre of each centrosome to a 1μm radius; $n_{\text{DSas-4-GFP}}$ = 8 centrosomes; $n_{\text{Asl-GFP}}$ = 24 centrosomes. Note that in our analysis of DSas-4-GFP the improved resolution allowed us to visualize centriole pairs at virtually all stages of the cell cycle. For ease of presentation, however, we present an image in Figure 1G where one centriole was below the plane of the other centriole, allowing us to obtain a maximum intensity profile through a single centriole; the average fluorescence intensity profile presented in Figure 1H was obtained from 8 such centrioles. This was not necessary for our analysis of Asl-GFP, as the ring of Asl-GFP was only detectable around one centriole (the mother) in each pair. Note that this super-resolution imaging leads to very rapid bleaching of DSas-4-GFP and Asl-GFP, so we could not perform high quality time lapse super-resolution imaging.

In all experiments, cell cycle stage was determined based on the timing of centrosome separation (indicating the start of *S*-phase) and nuclear envelope breakdown (NEB) (indicating the start of mitosis).

With the exception of Figure 1G,I all fluorescent images shown were obtained after 3D rendering of image data in Volocity, and the Volocity ‘smooth zoom filter’ was applied to all images. Images shown in Figure 1G,I are maximum intensity projections generated in Image J software with no filter applied.

Antibody injections. Anti-DSas-4 [5] or anti-Asl [1] antibodies used for immuno-inhibition experiments were covalently coupled to Texas Red as described previously [6]. The antibodies were injected into embryos at the onset of mitosis. Embryos were imaged immediately following antibody injection using the spinning disc confocal system described above. For FRAP experiments 3-4 old centrosomes were bleached in *S*-phase both on the side of the embryo closest to the injection site (antibody-blocked) and the side furthest form the injection site (control). Data shown was collated from at least 3 embryos for each experiment (see image analysis section above). We have shown previously that the injection of control IgG antibodies does not detectably interfere with the centrosome cycle or with any other aspect of embryo development [6], and have also shown examples where injected anti-centrosomal antibodies that were bound to centrosomes did not detectably perturb centrosome function [1].

Analysis of protein levels in whole embryo lysates. Embryos were dechorionated by bleach and then fixed in methanol containing 2mM EGTA, incubated in methanol at 4^0C for at least 24h, then re-hydrated in PBS+0.1% Triton X-100. 20 embryos were selected manually and homogenized in a total volume of 25µl SDS sample buffer. 10µl of each sample was separated on 3-8% NuPAGE acrylamide gels (Invitrogen) and then transferred to nitrocellulose membranes (0.2µm pore size, BIO-RAD). Membranes were blocked for 1h at room temperature in blocking solution (PBS+4% milk powder+0.1% Tween-20) before incubation with the primary antibody for 1h at room temperature (antibodies were diluted to 1-5ng/ml in blocking solution). Membranes were washed in PBS+0.1% Tween-20 and incubated with horseradish peroxidase-conjugated secondary antibody (GE Healthcare, diluted 3000-fold in blocking solution) for 45 minutes at room temperature. Following washing in PBS+0.1% Tween-20 membranes were incubated with enhanced chemiluminescent substrate for 1 minute (Thermo Scientific, #34095, diluted 2-5 -fold) and exposed to X-ray film.

SUPPLEMENTAL REFERENCES:

S1. Conduit, P.T., Brunk, K., Dobbelaere, J., Dix, C.I., Lucas, E.P., and Raff, J.W. (2010). Centrioles regulate centrosome size by controlling the rate of Cnn incorporation into the PCM. Curr Biol *20*, 2178-2186.

S2. Dzhindzhev, N.S., Yu, Q.D., Weiskopf, K., Tzolovsky, G., Cunha-Ferreira, I., Riparbelli, M., Rodrigues-Martins, A., Bettencourt-Dias, M., Callaini, G., and Glover, D.M. (2010). Asterless is a scaffold for the onset of centriole assembly. Nature *467*, 714-718.

S3. Blachon, S., Gopalakrishnan, J., Omori, Y., Polyanovsky, A., Church, A., Nicastro, D., Malicki, J., and Avidor-Reiss, T. (2008). Drosophila asterless and vertebrate Cep152 Are orthologs essential for centriole duplication. Genetics *180*, 2081-2094.

S4. Lucas, E.P., and Raff, J.W. (2007). Maintaining the proper connection between the centrioles and the pericentriolar matrix requires Drosophila centrosomin. J Cell Biol *178*, 725-732.

S5. Basto, R., Lau, J., Vinogradova, T., Gardiol, A., Woods, C.G., Khodjakov, A., and Raff, J.W. (2006). Flies without centrioles. Cell *125*, 1375-1386.

S6. Gergely, F., Kidd, D., Jeffers, K., Wakefield, J.G., and Raff, J.W. (2000). D-TACC: a novel centrosomal protein required for normal spindle function in the early Drosophila embryo. EMBO J *19*, 241-252.
