# Supplementary material for: Asterless Licenses Daughter Centrioles to Duplicate for the First Time in Drosophila Embryos
Source: Curr Biol. 2014 Jun 2;24(11):1276–82. doi: 10.1016/j.cub.2014.04.023 (PMC4046630; doi:10.1016/j.cub.2014.04.023)
Supplement: Document S2. Article plus Supplemental Information [file mmc2.pdf]

Current Biology 24, 1276–1282, June 2, 2014 ©2014 The Authors http://dx.doi.org/10.1016/j.cub.2014.04.023

Report

Asterless Licenses Daughter Centrioles to Duplicate for the First Time in *Drosophila* Embryos

Zsofia A. Novak,[1] Paul T. Conduit,[1] Alan Wainman,[1,2] and Jordan W. Raff[1,*]
[1]Sir William Dunn School of Pathology, University of Oxford, South Parks Road, Oxford OX1 3RE, UK
[2]Oxford Micron Advanced Bioimaging Unit, Department of Biochemistry, University of Oxford, South Parks Road, Oxford OX1 3QU, UK

Summary

Centrioles form centrosomes and cilia, and defects in any of these three organelles are associated with human disease [1]. Centrioles duplicate once per cell cycle, when a mother centriole assembles an adjacent daughter during S phase. Daughter centrioles cannot support the assembly of another daughter until they mature into mothers during the next cell cycle [2–5]. The molecular nature of this daughter-to-mother transition remains mysterious. Pioneering studies in *C. elegans* identified a set of core proteins essential for centriole duplication [6–12], and a similar set have now been identified in other species [10, 13–18]. The protein kinase ZYG-1/Sak/Plk4 recruits the inner centriole cartwheel components SAS-6 and SAS-5/Ana2/STIL, which then recruit SAS-4/CPAP, which in turn helps assemble the outer centriole microtubules [19, 20]. In flies and humans, the Asterless/Cep152 protein interacts with Sak/Plk4 and Sas-4/CPAP and is required for centriole duplication, although its precise role in the assembly pathway is unclear [21–24]. Here, we show that Asl is not incorporated into daughter centrioles as they assemble during S phase but is only incorporated once mother and daughter separate at the end of mitosis. The initial incorporation of Asterless (Asl) is irreversible, requires DSas-4, and, crucially, is essential for daughter centrioles to mature into mothers that can support centriole duplication. We therefore propose a "dual-licensing" model of centriole duplication, in which Asl incorporation provides a permanent primary license to allow new centrioles to duplicate for the first time, while centriole disengagement provides a reduplication license to allow mother centrioles to duplicate again.

Results

Daughter Centrioles Incorporate DSas-4, but Not Asl, during Their Assembly

To better understand how Asl and DSas-4 might function together in fly centriole duplication, we followed the behavior of GFP-fusions of these proteins in centrosomes during the rapid, early, mitotic cycles in living syncytial blastoderm *Drosophila* embryos. For all experiments, we expressed near-endogenous levels of either DSas-4-GFP or Asl-GFP [21] in the absence of the corresponding endogenous protein (Figures S1A and S1B available online).

In early S phase, just after the centrosomes have separated (Figure 1A, t = 0 s), the level of DSas-4-GFP fluorescence was similar at the two centrosomes and gradually increased during S phase, as new daughter centrioles assembled (Figures 1A and 1B). DSas-4-GFP levels plateaued shortly before the start of mitosis (nuclear envelope breakdown [NEB]; Figures 1A and 1B), when new daughter centrioles have reached their full size [25]; the fluorescence then steadily declined as mitosis proceeded. This behavior suggests that a pool of DSas-4 is stably incorporated into daughter centrioles as they form but that some "excess" DSas-4 is recruited during S phase and then lost during mitosis (Figure 1E). Fluorescence recovery after photobleaching (FRAP) experiments strongly supported this interpretation (Figures S1C–S1G).

Previous superresolution microscopy studies have shown that DSas-4 is tightly concentrated at centrioles in fly cells and does not spread into the pericentriolar paterial (PCM); it localizes within an outer ring of Asl that lies at the outer edge of the centrioles [26, 27]. We confirmed that this was also the case for the DSas-4-GFP and Asl-GFP fusion proteins in living embryos using 3D-structured illumination superresolution microscopy (Figures 1G–1I). Note that our superresolution images of DSas-4-GFP and Asl-GFP reveal the localization of the C terminus of both proteins, which are not predicted to colocalize: the C terminus of DSas-4 interacts with the N terminus of the centriole cartwheel protein Ana2 [28] and so would be predicted to lie internally to the C terminus of Asl, which is what we observe. Thus, we are confident that DSas-4-GFP is a bona fide marker of centrioles in these embryos.

In contrast to DSas-4, we observed dramatically different levels of Asl-GFP at the two separating centrioles in early S phase (Figure 1C, t = 0 s). An analysis with the centriole-age marker RFP-PACT [29] revealed that the centrosome that inherited the original mother centriole (hereafter the "old" centrosome) always exhibited more Asl-GFP than the centrosome that inherited the original daughter centriole (hereafter the "new" centrosome) (Figures S2A and S2B). Asl-GFP fluorescence in new centrosomes (Figures 1C and 1D, orange labels) steadily increased throughout S phase and into mitosis. Surprisingly, Asl-GFP fluorescence in the oldest centrosomes (Figures 1C and 1D, blue labels) did not appear to increase at all, even though these old centrosomes formed new daughters during this time. This strongly suggests that new daughter centrioles do not incorporate Asl-GFP while they assemble and that the incorporation of Asl-GFP we observe at new centrosomes (orange labels, Figures 1C and 1D) must be due to incorporation at the new mother centrioles (Figure 1F). We conclude that although Asl is essential for centriole duplication [21], Asl-GFP is surprisingly not incorporated into daughter centrioles as they assemble during S phase but only starts to be incorporated at about the time they separate from their mothers at the end of mitosis.

FRAP analysis of Asl-GFP at old centrosomes (where total GFP fluorescence levels are constant; Figures 1C and 1D) revealed that Asl-GFP fluorescence recovered after bleaching but plateaued at ~50% recovery (Figures S2C and S2D).

*Correspondence: jordan.raff@path.ox.ac.uk
This is an open access article under the CC BY license (http://creativecommons.org/licenses/by/3.0/).

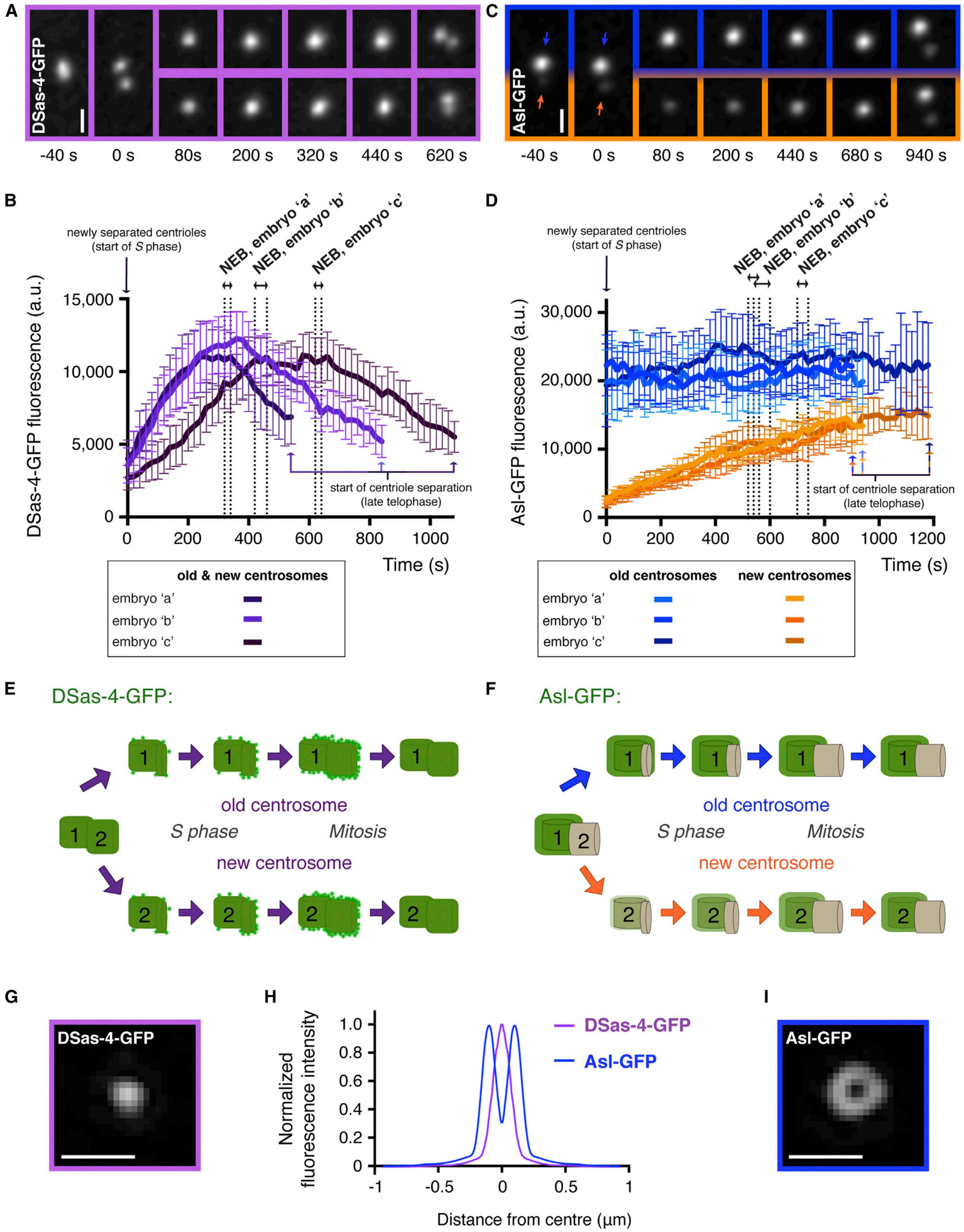

Figure 1. Daughter Centrioles Incorporate DSas-4, but Not Asl, during Their Assembly

(A) Fluorescence images from a time-lapse movie show DSas-4-GFP incorporation into newly separated centrosomes over a single cell cycle; time (s) relative to centriole separation at t = 0 s is indicated. Scale bar, 1 μm.

(legend continued on next page)

Thus, there are two fractions of Asl-GFP at mother centrosomes: ~50% appears to be irreversibly incorporated (hereafter the "immobile" fraction), while ~50% can still exchange with the cytosolic pool (hereafter the "mobile" fraction). Similar results were obtained for new centrosomes, but this analysis was complicated because new centrosomes also incorporate additional Asl-GFP protein during the cell cycle (Figures 1C and 1D and data not shown).

The Initial Incorporation of Asl into Newly Formed Centrioles Depends on DSas-4

The C-terminal region of Asl can interact with the N-terminal region of DSas-4 [22], and superresolution microscopy has revealed that these interacting domains precisely colocalize at the centriole wall [26]. We therefore tested whether DSas-4 might be required to recruit Asl to new centrioles at the end of mitosis.

We injected Texas-red-labeled, affinity-purified antibodies raised against the N-terminal region of DSas-4 (that should interfere with the binding of DSas-4 to Asl; Figure S3A) into embryos expressing Asl-GFP. This was done just as the embryos entered mitosis, when daughter centrioles had fully formed [25] (and so had incorporated DSas-4) but had not yet separated from their mothers (and so had not yet incorporated Asl). The antibodies bound to the centrioles that were close to the injection site (Figure 2A, DSas-4 blocked; Figure S3B), but not to centrioles that were further away from the injection site (Figure 2A, "control"; Figure S3B). By the time the centrioles had separated at the start of S phase (Figures 2B and S3C), Asl-GFP localized to both old and new centrosomes in the control region but failed to localize to the new centrosomes in the DSas-4-antibody-blocked region (Figures 2B and 2C); importantly, new centrosomes were decorated with anti-DSas-4 antibodies, indicating that new centrosomes were present and the new mother centrioles in these centrosomes had successfully incorporated DSas-4. We conclude that DSas-4 is incorporated into new centrioles before Asl and that the anti-DSas-4 antibodies block the interaction between DSas-4 and Asl, and so block the subsequent incorporation of Asl into new centrioles at the end of mitosis (see schematic illustration, Figure 2B). We cannot formally exclude the unlikely possibility, however, that the anti-DSas-4 antibodies block the incorporation of Asl into new centrioles by a mechanism that does not depend on their blocking the interaction of DSas-4 with Asl.

We noticed that the anti-DSas-4 antibodies did not detectably perturb the localization of Asl-GFP in old centrosomes, even though their centrioles were decorated with the antibodies (Figure 2B). FRAP experiments indicated that the mobile fraction of Asl-GFP in old centrosomes continued to turn over with near-normal kinetics, despite the presence of the antibodies (Figure S3D). Thus, although anti-DSas-4 antibodies successfully block the initial recruitment of Asl to new centrioles, they do not block either the recruitment of the mobile Asl fraction to old centrioles or the maintenance of the immobile Asl fraction there.

Asl Incorporation Allows Newly Assembled, Disengaged Centrioles To Duplicate for the First Time

Asl is essential for centriole duplication in flies [21, 30], so we reasoned that its incorporation into newly assembled centrioles might be required to allow them to mature into mothers competent for duplication in the next cell cycle. To investigate this possibility, we needed to specifically block the incorporation of Asl into newly disengaged daughter centrioles. As just described, injections of anti-DSas-4 antibodies did block this incorporation (Figures 2A–2C), and we showed previously that these antibodies also block centriole duplication [17]. Because DSas-4 itself is essential for centriole duplication, however, we could not be sure that it was the lack of Asl incorporation into new centrioles that was blocking their subsequent duplication. We needed to block Asl incorporation without directly interfering with DSas-4 function.

We therefore used Texas-red-labeled, affinity-purified antibodies raised against the C-terminal region of Asl, which should interfere with its binding to DSas-4 (Figure S3A). We injected the antibodies into DSas-4-GFP-expressing embryos that were entering mitosis. The antibodies bound to centrosomes close to the injection site (Figure 2D), and, as the centrosomes separated at the end of mitosis, DSas-4-GFP fluorescence was localized to both old and new centrosomes, indicating that the antibodies did not interfere with the localization of DSas-4-GFP molecules that had already been incorporated into the centrioles at the time of the antibody injection (Figures 2E and 2F). We noticed, however, that the anti-Asl antibodies often decorated only one centrosome of the separating pair (Figure 2E)—presumably the old centrosome, because it contains the original mother centriole that would have already incorporated Asl at the time of the antibody injection. This finding suggests that the antibodies bind to Asl molecules in the mother centrioles but also to Asl molecules in the cytoplasm, thereby blocking their incorporation into the newly assembled centrioles (see schematic illustration, Figure 2E).

To test whether these newly separated centrioles that lacked Asl could support centriole duplication, we compared the incorporation of the centriole marker DSas-4-GFP at old centrosomes (that have inherited an old mother centriole containing both DSas-4 and Asl) and at new centrosomes (that have inherited a new mother centriole containing DSas-4, but not Asl [arrowhead, Figure 2E]). Despite being heavily decorated with anti-Asl antibodies, old centrosomes incorporated additional DSas-4-GFP, indicating that mother centrioles were assembling new daughters (Figures 3 and S3F, blue

(B) Graph shows averaged centrosomal DSas-4-GFP fluorescence (a.u. = arbitrary units) over time from three embryos (>25 centrosomes analyzed in each). Error bars indicate the SD.

(C and D) Images (C) and graph (D) show Asl-GFP incorporation into newly separated centrosomes, presented as in (A) and (B), respectively. Note that new centrosomes (orange box and graph) have not reached as high a level of fluorescence as the old centrosomes (blue box and graph) by the end of the cycle; this is because new centrosomes continue to incorporate some Asl-GFP in the following cell cycle (Figure S2E). For this reason, the analysis of old centrosomes shown here focuses on older (i.e., brighter) centrosomes that had already reached their full brightness (see Figure S2E legend for more detail).

(E and F) Schematic interpretation of how DSas-4-GFP (E) and Asl-GFP (F) incorporate into centrioles.

(G–I) Three-dimensional structured illumination microscopy (3D-SIM) superresolution images of centriolar DSas-4-GFP (G) or Asl-GFP (I) in living embryos (see the Supplemental Experimental Procedures for a full explanation of how these data were obtained and analyzed). Scale bars, 0.5 μm. (H) Graph shows the average centriolar fluorescence intensity profiles of DSas-4-GFP (purple, n = 8) and Asl-GFP (blue, n = 24) in 3D-SIM images. Note that in (E), the immobile fraction of DSas-4-GFP is shown associated with the centrioles, while, for simplicity, the mobile fraction (that subsequently dissociates during mitosis) is shown tightly surrounding the centrioles based on our localization data (G and H).

See also Figures S1 and S2 and the Supplemental Experimental Procedures.

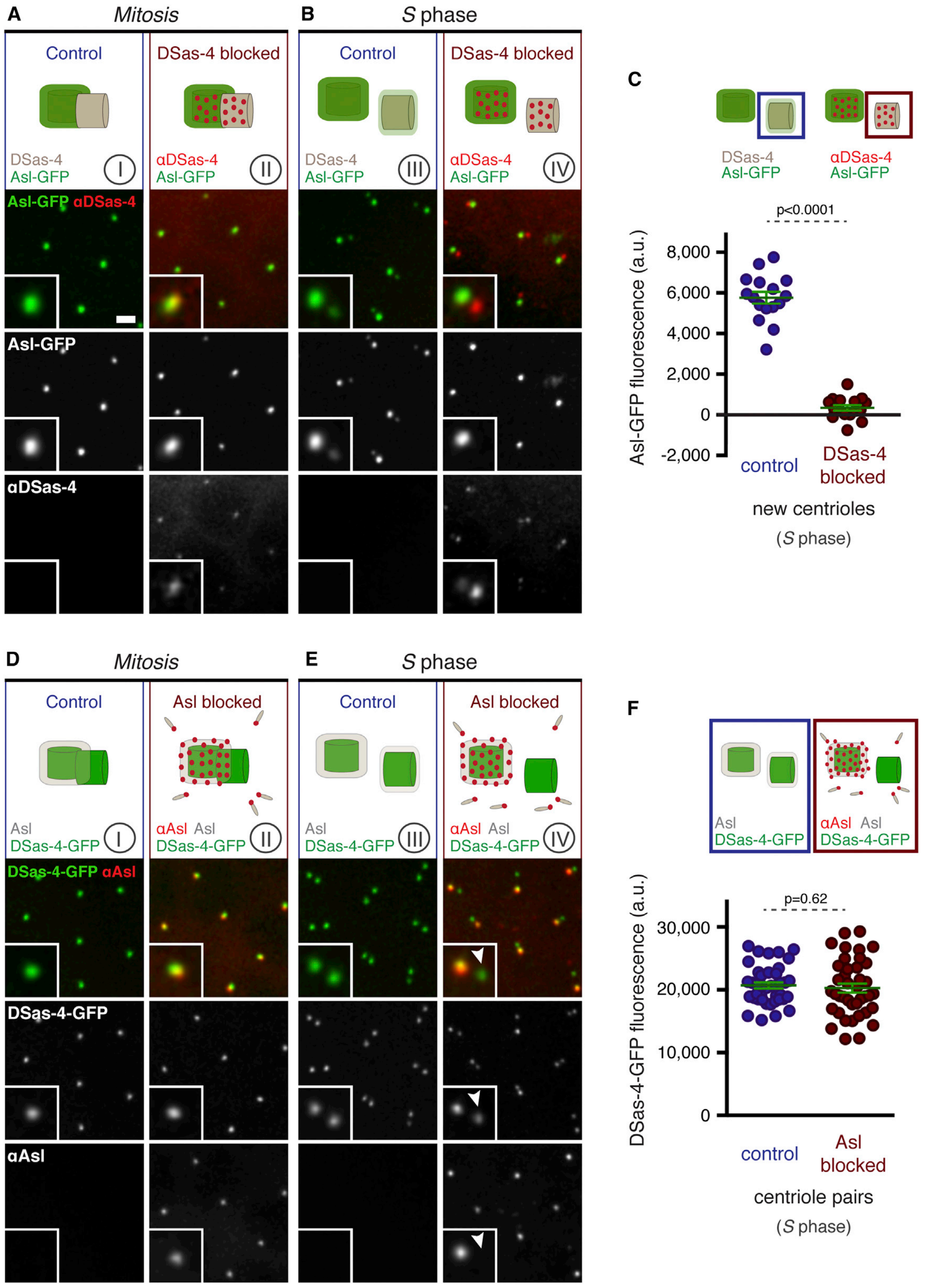

Figure 2. The Initial Incorporation of Asl-GFP into Newly Assembled Centrioles Can Be Inhibited with Anti-DSas-4 or Anti-Asl Antibodies

(A and B) Fluorescence images show two regions ("control" and "DSas-4-blocked") of an embryo expressing Asl-GFP (green) that has been injected with Texas-red-labeled anti-DSas-4 antibodies (red) at the start of mitosis (columns I and II) and several minutes later after the centrioles have separated at the start of S phase (columns III and IV). The control region (columns I and III) is far from the injection site (see Figures S3B and S3C), so no antibodies are detectable; the DSas-4-blocked region (columns II and IV) is close to the injection site, and the antibodies bind to the centrioles. The schematic at the top of each panel illustrates how the DSas-4 antibodies bind to centrioles in mitosis (II) and block the subsequent incorporation of Asl-GFP into the new centriole at the start of S phase, but they do not interfere with Asl-GFP localization at the old centriole (IV).

(legend continued on next page)

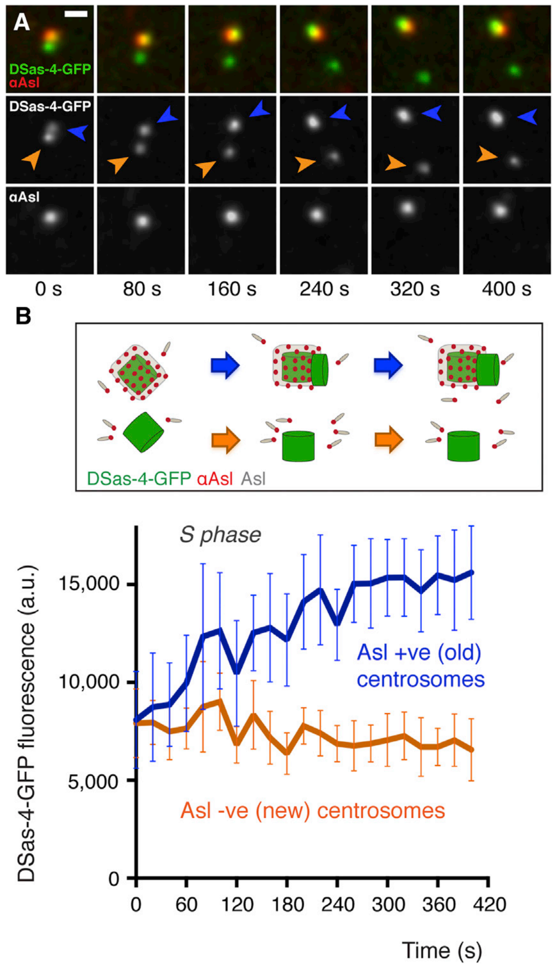

Figure 3. Asl Recruitment to New Centrioles Is Required for Their Duplication

(A) Fluorescence images from a time-lapse movie of an embryo expressing DSas-4-GFP (green) that has been injected with Texas-red-labeled anti-Asl antibodies (red); time (s) relative to centriole separation at t = 0 s is indicated. DSas-4-GFP is incorporated into newly separated old centrosomes (blue arrowheads), even though these centrioles are strongly decorated with anti-Asl antibodies. DSas-4-GFP is not, however, incorporated into newly separated young centrosomes (orange arrowheads), even though no anti-Asl antibodies are detectably binding to these centrosomes. Scale bar, 1 μm.
(B) Graph (with explanatory schematic) quantifies the incorporation of DSas-4-GFP into old anti-Asl-decorated centrosomes (blue line); there is no incorporation into young Asl-negative centrosomes (orange line). Error bars indicate the SEM.
See also Figure S3 and the Supplemental Experimental Procedures.

labels). In contrast, even though new centrosomes were not detectably decorated with anti-Asl antibodies, they did not incorporate any additional DSas-4-GFP, indicating that these new mother centrioles could not assemble daughters (Figures 3 and S3F, orange labels). Thus, new centrioles that lack Asl appear unable to initiate centriole duplication, even though they have disengaged from their mothers and have passed through mitosis.

Discussion

Our observations demonstrate that Asl recruitment to disengaged new centrioles has a critical role in allowing these centrioles to mature into mothers that can duplicate for the first time. During all subsequent duplication cycles, however, mother centrioles already contain a pool of immobile Asl, and this appears to be sufficient to allow subsequent rounds of duplication, because anti-Asl antibodies block the recruitment of the mobile fraction of Asl to mother centrioles (Figure S3E) but do not block their duplication (Figure 3). For an old centriole to duplicate again, therefore, disengagement of the daughter centriole appears to be the crucial licensing event that allows reduplication [4, 5, 31], because immobile Asl incorporation has already occurred. Taken together, our findings suggest a dual-licensing model in which the recruitment of the immobile fraction of Asl by DSas-4 provides an irreversible primary license to allow newly formed centrioles to duplicate for the first time, while centriole disengagement provides a reduplication license [5] to allow older centrioles to duplicate again (Figure 4).

How might Asl perform this primary licensing function? In flies, Asl localizes Sak to centrioles [22], probably explaining why Asl incorporation is a crucial step in converting a disengaged daughter centriole into a mother centriole that can duplicate. Cep152 (human Asl) is also required for the efficient loading of Plk4 (human Sak) onto centrioles in vertebrate cells [22–24], although it appears to share this function with Cep192 (human SPD-2) [32, 33]. Our model is consistent with superresolution microscopy studies on fixed cells, which show that Asl/Cep152 is associated with the mother centriole in an engaged centriole pair [27, 32, 34, 35], suggesting that a similar model may operate in vertebrates. Although the primary and reduplication licensing steps are mechanistically different, we suspect that they share a common purpose: to provide an Asl platform that is competent to recruit Sak to initiate daughter centriole assembly (Figure 4).

Our model can explain why only mother centrioles can support certain types of experimentally induced centriole reduplication, including that induced by Sak overexpression [2, 3] or by ablation of one of the engaged centrioles during an arrested S phase [4]. It can also explain why daughter centrioles appear to have to be "modified" before they can support any duplication [5]; our results strongly suggest that this modification, at least in flies, is Asl incorporation.

How is Asl recruited to centrioles? We speculate that DSas-4 initially recruits the immobile fraction of Asl, which

(C) The graph quantifies Asl-GFP levels in new centrioles in early S phase in the control region and in the DSas-4-blocked region (n = 16 centrioles from three injected embryos), as shown schematically above the graph. Error bars indicate the SEM.
(D and E) Fluorescence images show two regions of an embryo expressing DSas-4-GFP (green) injected with Texas-red-labeled anti-Asl antibodies (red), presented as in (A) and (B) above. The schematics illustrate how the anti-Asl antibodies do not perturb the localization of DSas-4-GFP that is already incorporated into the centrioles at the time of antibody injection but bind the endogenous (nonfluorescent) Asl molecules (gray) in the cytoplasm and so block their incorporation into the new centriole.
(F) The graph quantifies DSas-4-GFP levels in centriole pairs in early S phase in either control or Asl-blocked regions (n = 40 centriole pairs from four embryos), as illustrated schematically above the graph. Note that we compare centriole pairs instead of individual centrioles in this experiment because we cannot distinguish old and young control centrioles based on DSas-4-GFP levels alone. Scale bars, 2 μm. Error bars indicate the SEM.
See also Figure S3 and the Supplemental Experimental Procedures.

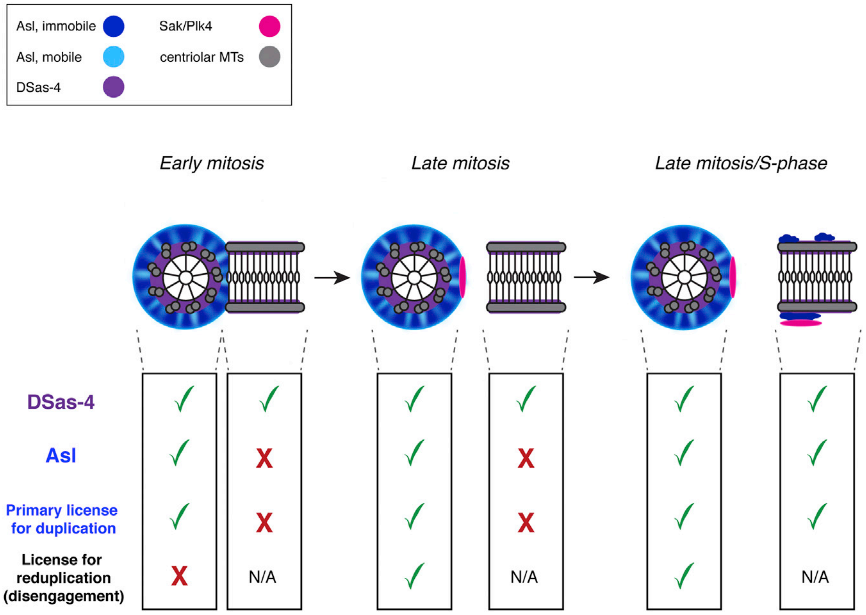

Figure 4. A Dual Licensing Model of Centriole Duplication

The schematic diagrams illustrate a centriole pair, with mother in end-on view (left) and daughter in side-on view (right), as they pass through mitosis. The table below illustrates the state of each centriole in terms of DSas-4 incorporation, Asl incorporation, and whether the centriole has a primary license for its first duplication or a reduplication license for subsequent rounds of duplication. In early mitosis, the mother centriole has incorporated immobile Asl during a previous cell cycle, which irreversibly provided it with a primary license, but it is unable to duplicate again because it lacks a reduplication license, which it will acquire when it disengages from its daughter [4, 5, 37]. The daughter centriole has incorporated DSas-4, but not Asl, and so it lacks a primary license and cannot duplicate until it disengages and matures into a mother. In late mitosis, the centrioles disengage: the mother centriole thereby acquires a reduplication license (speculatively illustrated here by a free “patch” of Sak on its side); the separated daughter centriole cannot duplicate until it starts to incorporate Asl (shown here occurring in late mitosis/S phase), which allows it to recruit Sak for the first time. In *Drosophila* blastoderm embryos, Asl incorporation starts around the time centrioles disengage at the end of mitosis; because centriole disengagement is also closely linked to S phase initiation, we cannot tell whether Asl incorporation is triggered by centriole disengagement or by S phase initiation.

then recruits the mobile fraction. This would explain the 50:50 ratio of immobile to mobile Asl (Figures S2C, S2D, S3D, and S3E). Our finding that anti-Asl antibodies strongly block the recruitment of the mobile fraction of Asl to mother centrosomes (Figure S3E) also supports this possibility. It is tempting to speculate that the mobile fraction of Asl may be important for the previously described role of Asl in mitotic PCM recruitment [22, 29, 36]. It is also interesting to note that only very low levels of Asl seem to be required at new mother centrioles to allow duplication (Figures 1C and 1D).

It remains to be determined what regulates the interaction between DSas-4 and Asl such that Asl is only recruited to daughter centrioles at about the time they separate from their mothers. We speculate that the phosphorylation state of either or both proteins could be altered at the end of mitosis, perhaps increasing the affinity of their interaction. Polo/Plk1 seems to play a crucial part in resetting the reduplication license at old centrioles through the regulation of centriole disengagement [37]; perhaps it also has an important role in the primary licensing of new centrioles by regulating the interaction between DSas-4 and Asl.

Supplemental Information

Supplemental Information includes three figures and Supplemental Experimental Procedures and can be found with this article online at http://dx.doi.org/10.1016/j.cub.2014.04.023.

Acknowledgments

We thank Ian Dobbie, Lothar Schermelleh, and Justin Demmerle from Micron Oxford for help and advice on live 3D structured illumination microscopy imaging. We also thank members of the J.W.R. laboratory and Francis Barr for advice and comments on the manuscript, Saroj Saurya for technical assistance, and Tomer Avidor-Reiss for the Asl-GFP *Drosophila* line. The research was funded by a Wellcome Trust PhD studentship (to Z.A.N.), a Cancer Research UK program grant (C5395/A10530) (to P.T.C., A.W., and J.W.R.), and a Wellcome Trust strategic award (091911) to the Micron Oxford Advanced Bioimaging Unit (for A.W.).

Received: December 22, 2013
Revised: March 11, 2014
Accepted: April 11, 2014
Published: May 15, 2014

References

1. Nigg, E.A., and Raff, J.W. (2009). Centrioles, centrosomes, and cilia in health and disease. Cell *139*, 663–678.
2. Kleylein-Sohn, J., Westendorf, J., Le Clech, M., Habedanck, R., Stierhof, Y.D., and Nigg, E.A. (2007). Plk4-induced centriole biogenesis in human cells. Dev. Cell *13*, 190–202.
3. Cunha-Ferreira, I., Rodrigues-Martins, A., Bento, I., Riparbelli, M., Zhang, W., Laue, E., Callaini, G., Glover, D.M., and Bettencourt-Dias, M. (2009). The SCF/Slimb ubiquitin ligase limits centrosome amplification through degradation of SAK/PLK4. Curr. Biol. *19*, 43–49.
4. Loncarek, J., Hergert, P., Magidson, V., and Khodjakov, A. (2008). Control of daughter centriole formation by the pericentriolar material. Nat. Cell Biol. *10*, 322–328.
5. Wang, W.J., Soni, R.K., Uryu, K., and Tsou, M.F. (2011). The conversion of centrioles to centrosomes: essential coupling of duplication with segregation. J. Cell Biol. *193*, 727–739.
6. Pelletier, L., Ozlü, N., Hannak, E., Cowan, C., Habermann, B., Ruer, M., Müller-Reichert, T., and Hyman, A.A. (2004). The Caenorhabditis elegans centrosomal protein SPD-2 is required for both pericentriolar material recruitment and centriole duplication. Curr. Biol. *14*, 863–873.
7. Kemp, C.A., Kopish, K.R., Zipperlen, P., Ahringer, J., and O’Connell, K.F. (2004). Centrosome maturation and duplication in C. elegans require the coiled-coil protein SPD-2. Dev. Cell *6*, 511–523.
8. O’Connell, K.F., Caron, C., Kopish, K.R., Hurd, D.D., Kemphues, K.J., Li, Y., and White, J.G. (2001). The C. elegans zyg-1 gene encodes a regulator of centrosome duplication with distinct maternal and paternal roles in the embryo. Cell *105*, 547–558.
9. Dammermann, A., Müller-Reichert, T., Pelletier, L., Habermann, B., Desai, A., and Oegema, K. (2004). Centriole assembly requires both centriolar and pericentriolar material proteins. Dev. Cell *7*, 815–829.
10. Leidel, S., Delattre, M., Cerutti, L., Baumer, K., and Gönczy, P. (2005). SAS-6 defines a protein family required for centrosome duplication in C. elegans and in human cells. Nat. Cell Biol. *7*, 115–125.
11. Delattre, M., Leidel, S., Wani, K., Baumer, K., Bamat, J., Schnabel, H., Feichtinger, R., Schnabel, R., and Gönczy, P. (2004). Centriolar SAS-5

is required for centrosome duplication in C. elegans. Nat. Cell Biol. *6*, 656–664.
12. Leidel, S., and Gönczy, P. (2003). SAS-4 is essential for centrosome duplication in C elegans and is recruited to daughter centrioles once per cell cycle. Dev. Cell *4*, 431–439.
13. Bettencourt-Dias, M., Rodrigues-Martins, A., Carpenter, L., Riparbelli, M., Lehmann, L., Gatt, M.K., Carmo, N., Balloux, F., Callaini, G., and Glover, D.M. (2005). SAK/PLK4 is required for centriole duplication and flagella development. Curr. Biol. *15*, 2199–2207.
14. Habedanck, R., Stierhof, Y.D., Wilkinson, C.J., and Nigg, E.A. (2005). The Polo kinase Plk4 functions in centriole duplication. Nat. Cell Biol. *7*, 1140–1146.
15. Stevens, N.R., Dobbelaere, J., Brunk, K., Franz, A., and Raff, J.W. (2010). Drosophila Ana2 is a conserved centriole duplication factor. J. Cell Biol. *188*, 313–323.
16. Arquint, C., Sonnen, K.F., Stierhof, Y.D., and Nigg, E.A. (2012). Cell-cycle-regulated expression of STIL controls centriole number in human cells. J. Cell Sci. *125*, 1342–1352.
17. Basto, R., Lau, J., Vinogradova, T., Gardiol, A., Woods, C.G., Khodjakov, A., and Raff, J.W. (2006). Flies without centrioles. Cell *125*, 1375–1386.
18. Hung, L.Y., Tang, C.J., and Tang, T.K. (2000). Protein 4.1 R-135 interacts with a novel centrosomal protein (CPAP) which is associated with the gamma-tubulin complex. Mol. Cell. Biol. *20*, 7813–7825.
19. Pelletier, L., O'Toole, E., Schwager, A., Hyman, A.A., and Müller-Reichert, T. (2006). Centriole assembly in Caenorhabditis elegans. Nature *444*, 619–623.
20. Delattre, M., Canard, C., and Gönczy, P. (2006). Sequential protein recruitment in C. elegans centriole formation. Curr. Biol. *16*, 1844–1849.
21. Blachon, S., Gopalakrishnan, J., Omori, Y., Polyanovsky, A., Church, A., Nicastro, D., Malicki, J., and Avidor-Reiss, T. (2008). Drosophila asterless and vertebrate Cep152 Are orthologs essential for centriole duplication. Genetics *180*, 2081–2094.
22. Dzhindzhev, N.S., Yu, Q.D., Weiskopf, K., Tzolovsky, G., Cunha-Ferreira, I., Riparbelli, M., Rodrigues-Martins, A., Bettencourt-Dias, M., Callaini, G., and Glover, D.M. (2010). Asterless is a scaffold for the onset of centriole assembly. Nature *467*, 714–718.
23. Cizmecioglu, O., Arnold, M., Bahtz, R., Settele, F., Ehret, L., Haselmann-Weiss, U., Antony, C., and Hoffmann, I. (2010). Cep152 acts as a scaffold for recruitment of Plk4 and CPAP to the centrosome. J. Cell Biol. *191*, 731–739.
24. Hatch, E.M., Kulukian, A., Holland, A.J., Cleveland, D.W., and Stearns, T. (2010). Cep152 interacts with Plk4 and is required for centriole duplication. J. Cell Biol. *191*, 721–729.
25. Callaini, G., and Riparbelli, M.G. (1990). Centriole and centrosome cycle in the early Drosophila embryo. J. Cell Sci. *97*, 539–543.
26. Mennella, V., Keszthelyi, B., McDonald, K.L., Chhun, B., Kan, F., Rogers, G.C., Huang, B., and Agard, D.A. (2012). Subdiffraction-resolution fluorescence microscopy reveals a domain of the centrosome critical for pericentriolar material organization. Nat. Cell Biol. *14*, 1159–1168.
27. Fu, J., and Glover, D.M. (2012). Structured illumination of the interface between centriole and peri-centriolar material. Open Biol. *2*, 120104.
28. Cottee, M.A., Muschalik, N., Wong, Y.L., Johnson, C.M., Johnson, S., Andreeva, A., Oegema, K., Lea, S.M., Raff, J.W., and van Breugel, M. (2013). Crystal structures of the CPAP/STIL complex reveal its role in centriole assembly and human microcephaly. Elife *2*, e01071.
29. Conduit, P.T., Brunk, K., Dobbelaere, J., Dix, C.I., Lucas, E.P., and Raff, J.W. (2010). Centrioles regulate centrosome size by controlling the rate of Cnn incorporation into the PCM. Curr. Biol. *20*, 2178–2186.
30. Dobbelaere, J., Josué, F., Suijkerbuijk, S., Baum, B., Tapon, N., and Raff, J. (2008). A genome-wide RNAi screen to dissect centriole duplication and centrosome maturation in Drosophila. PLoS Biol. *6*, e224.
31. Tsou, M.F., and Stearns, T. (2006). Mechanism limiting centrosome duplication to once per cell cycle. Nature *442*, 947–951.
32. Sonnen, K.F., Gabryjonczyk, A.M., Anselm, E., Stierhof, Y.D., and Nigg, E.A. (2013). Human Cep192 and Cep152 cooperate in Plk4 recruitment and centriole duplication. J. Cell Sci. *126*, 3223–3233.
33. Kim, T.S., Park, J.E., Shukla, A., Choi, S., Murugan, R.N., Lee, J.H., Ahn, M., Rhee, K., Bang, J.K., Kim, B.Y., et al. (2013). Hierarchical recruitment of Plk4 and regulation of centriole biogenesis by two centrosomal scaffolds, Cep192 and Cep152. Proc. Natl. Acad. Sci. USA *110*, E4849–E4857.
34. Sir, J.H., Barr, A.R., Nicholas, A.K., Carvalho, O.P., Khurshid, M., Sossick, A., Reichelt, S., D'Santos, C., Woods, C.G., and Gergely, F. (2011). A primary microcephaly protein complex forms a ring around parental centrioles. Nat. Genet. *43*, 1147–1153.
35. Lukinavičius, G., Lavogina, D., Orpinell, M., Umezawa, K., Reymond, L., Garin, N., Gönczy, P., and Johnsson, K. (2013). Selective chemical crosslinking reveals a Cep57-Cep63-Cep152 centrosomal complex. Curr. Biol. *23*, 265–270.
36. Varmark, H., Llamazares, S., Rebollo, E., Lange, B., Reina, J., Schwarz, H., and Gonzalez, C. (2007). Asterless is a centriolar protein required for centrosome function and embryo development in Drosophila. Curr. Biol. *17*, 1735–1745.
37. Tsou, M.F., Wang, W.J., George, K.A., Uryu, K., Stearns, T., and Jallepalli, P.V. (2009). Polo kinase and separase regulate the mitotic licensing of centriole duplication in human cells. Dev. Cell *17*, 344–354.

Current Biology, Volume *24*

Supplemental Information

Asterless Licenses Daughter Centrioles to Duplicate for the First Time in *Drosophila* Embryos

Zsofia A. Novak, Paul T. Conduit, Alan Wainman, and Jordan W. Raff

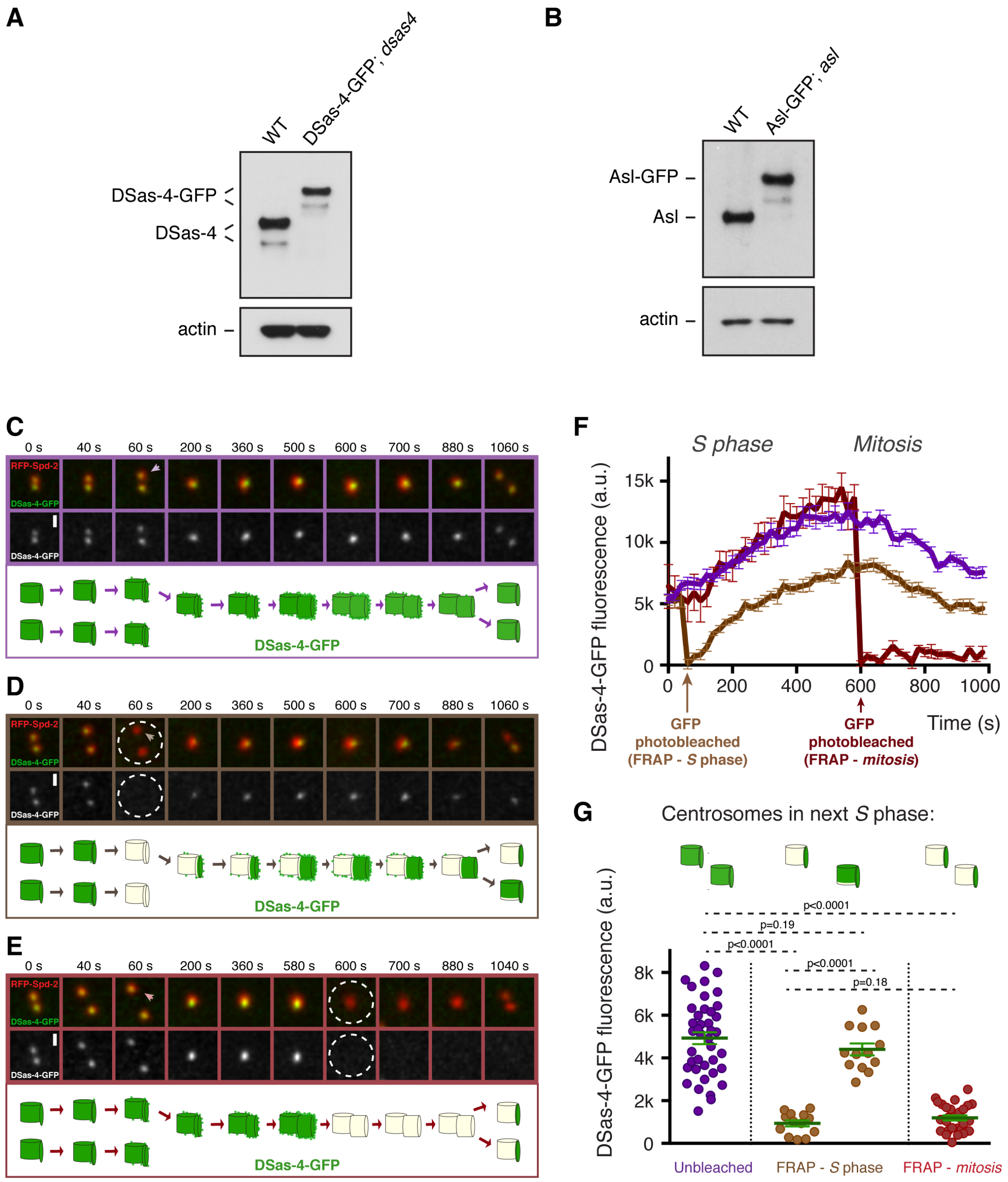

Figure S1. DSas-4-GFP is stably incorporated into growing centrioles during *S*-phase and excess DSas-4-GFP is lost from centrioles during mitosis (related to Figure 1). (A,B) Western blot analyses of embryo extracts shows that, in the embryos used for our analyses, DSas-4-GFP (A) and Asl-GFP (B) are expressed at similar levels to the corresponding endogenous protein in wild-type embryos. Representative blots from 3 independent repeats of each experiment are shown. (C-E) Fluorescence images from time-lapse FRAP movies show DSas-4-GFP (*green* in merged panels)

incorporation into newly separated (t=0s) control (non-bleached) centrosomes (C), centrosomes that were bleached in early *S*-phase (t=60s) (D), or centrosomes that were bleached in mitosis (t=600s) (E). The arrows at t=60s identify the centrosome from the separating pair that was followed in the subsequent panels. These embryos also expressed the centrosome marker RFP-DSpd-2 (*red*) so that centrioles could be followed even after photobleaching. A schematic interpretation of the distribution of DSas-4-GFP at each time point is shown below each set of fluorescence panels. Scale bars: 1μm. (F) Graph shows the quantification through time of DSas-4-GFP fluorescence levels at non-bleached centrosomes (*purple*, n=8 centrosomes from 2 embryos), centrosomes bleached in *S*-phase (*brown*, n=7 centrosomes from 2 embryos) and centrosomes bleached in mitosis (*red*, n=4 centrosomes from 2 embryos). The colours of the lines match the colours of the boxes surrounding the images shown in (C-E). Error bars represent s.e.m. Note how the centrosomes bleached in *S*-phase (*brown* line) show a very similar pattern of incorporation as the control centrosomes (*purple* line), although they are less bright, presumably because the DSas-4-GFP incorporated into the mother centriole does not turn over, and so its fluorescence has been irreversibly bleached (as depicted in the cartoon in (D)). This interpretation is supported by the observation that the centrosomes bleached in mitosis do not detectably incorporate DSas-4-GFP during mitosis. (G) Quantification of centrosomal DSas-4-GFP fluorescence at the start of the next *S*-phase for centrosomes that were unbleached (*purple*, n = 40 centrosomes from 4 embryos), bleached in the previous *S*-phase (*brown*, n = 26 centrosomes from 4 embryos), or bleached in the previous mitosis (red, n = 28 centrosomes from 4 embryos). Error bars represent s.e.m. Note how centrosomes bleached in the previous *S*-phase are highly asymmetric in fluorescence, presumably due to the irreversible bleaching of the mother centriole in *S*-phase.

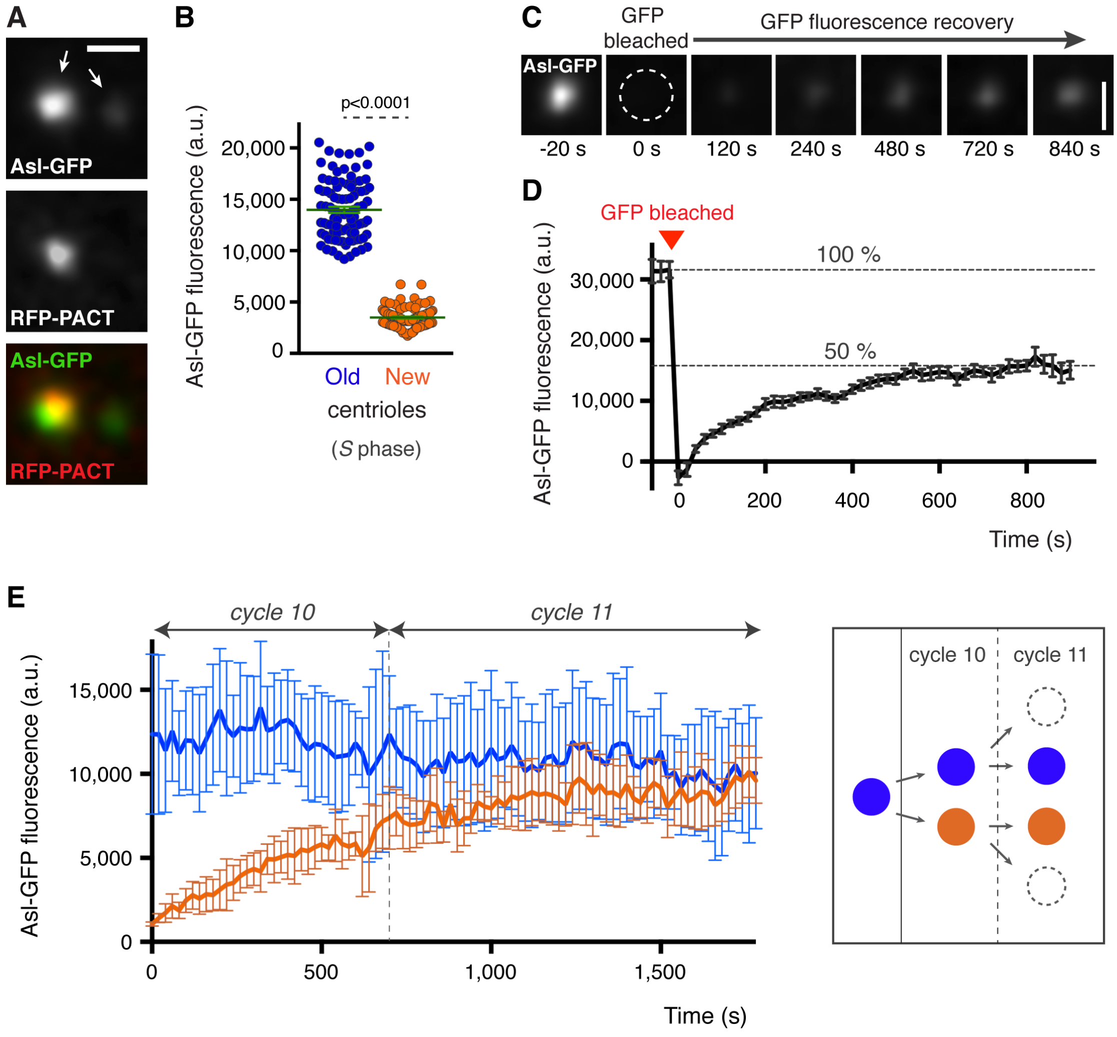

Figure S2. Further characterization of Asl-GFP behavior in living embryos (related to Figure 1).

(A) Images show the levels of Asl-GFP and RFP-PACT in two newly separated centrosomes in early *S*-phase. RFP-PACT fluorescence preferentially marks the older centriole [1], confirming that the centrosome with the older centriole contains more Asl-GFP. Scale bar: 1µm. (B) Graph quantifying Asl-GFP fluorescence levels at old and new centrioles (n=95 centriole pairs from 4 embryos). The old and new centrioles were distinguished based on RFP-PACT fluorescence. Statistical significance was calculated using a paired two-tailed t-test. Error bars represent s.e.m. (C,D) Images (C) and graph (D) show Asl-GFP fluorescence recovery following photobleaching of the Asl-GFP signal at old centrosomes at the start of *S*-phase. Time relative to photobleaching at t=0s is shown under each image. Scale bar: 1µm. Note how Asl-

GFP fluorescence recovers to only ~50% of the original pre-bleached levels; as total Asl-GFP fluorescence levels are not increasing at these centrosomes (Figure 1D, *blue* line), this indicates that ~50% of the Asl-GFP is turning over, while ~50% is immobile. In (D), the averaged fluorescence of 17 centrosomes from 3 embryos is shown. Error bars represent s.e.m. (E) Graph shows the averaged centrosomal Asl-GFP fluorescence (a.u.) over two complete nuclear cycles in a representative embryo (n=7 centrosomes each for old and new centrosomes). Error bars represent s.d. Multiple embryos were analyzed and they each showed the same qualitative result, a single embryo is shown for clarity. The graph illustrates how the old centrosomes (*blue*) maintain a relatively constant level of fluorescence over time, while the new centrosomes (*orange*), gradually accumulate fluorescence over two cycles; fluorescence levels at these new centrosomes plateau at approximately the same level as the old centrosomes towards the end of the second cycle. Because new centrosomes incorporate Asl-GFP over two cycles, if we simply compare the incorporation of Asl at *all* old and new centrosomes (in an unbiased manner), then the old centrosomes, on average, slightly increase in fluorescence due to the incorporation of Asl-GFP into the old centrosomes that are only one cycle old. For this reason, we only compare Asl-GFP levels in new centrosomes and the brightest (i.e., oldest) old centrosomes in Figure 1D (and here in Figure S2E), as these oldest centrosomes have stopped incorporating Asl-GFP. This analysis most clearly reveals that no Asl-GFP is being incorporated into the newly forming daughter centrioles, as the analysis is not complicated by the low level of incorporation into mother centrioles that are only one cell cycle old. Note that, although photobleaching is corrected for in these analyses, the centrosomes get slightly dimmer during the extended time course of these experiments (most easily visualized by the slow decline in fluorescence of the oldest centrosomes (*blue*) in (E)). This is a consistent feature that we see in extended time course experiments with many GFP-fusion proteins, and we suspect it occurs because the autofluorescence of the embryo bleaches more slowly than the GFP fluorescence; thus, our correction for photobleaching (which is based on photobleaching of the whole embryo) slightly under-corrects for the photobleaching of GFP.

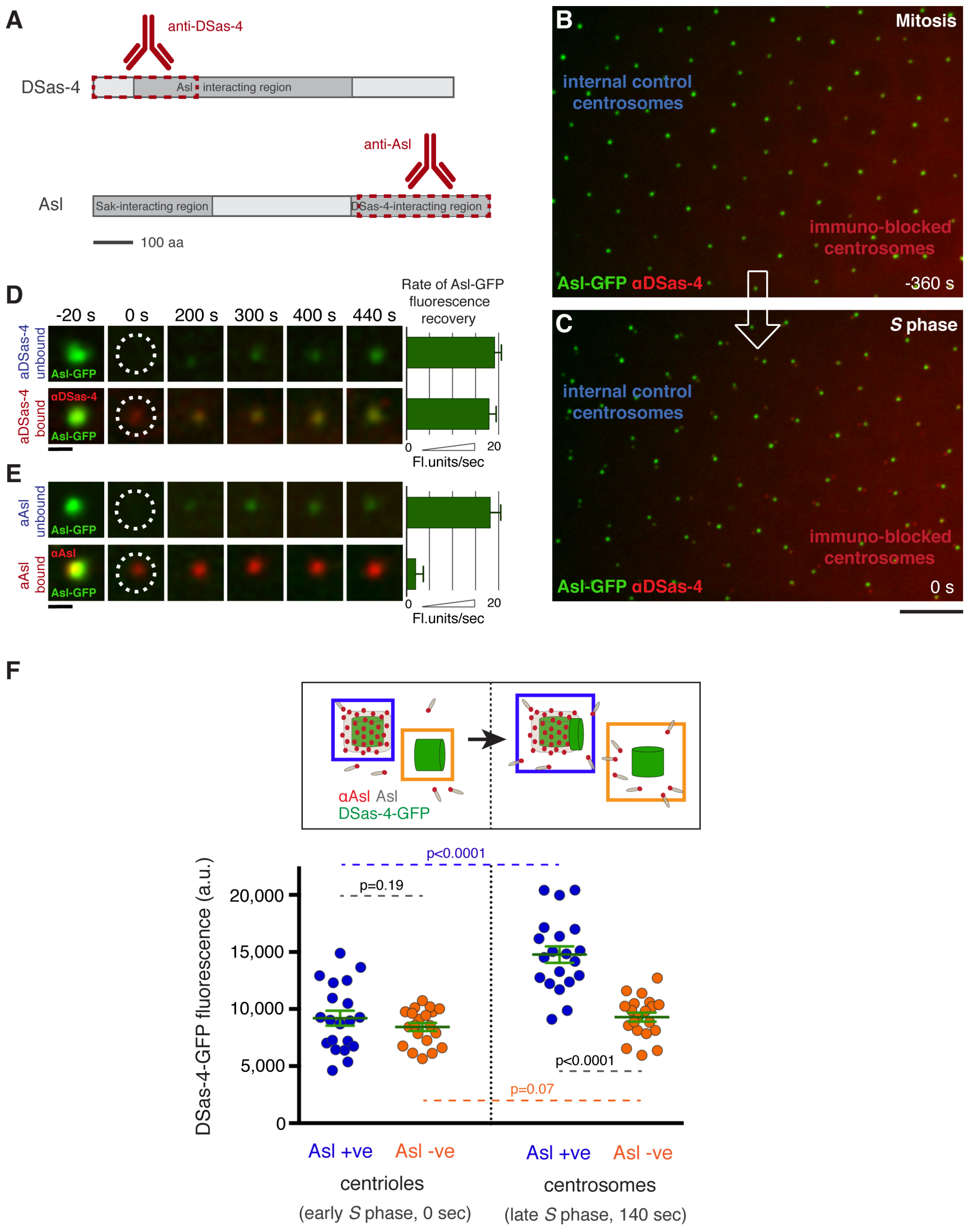

Figure S3. Multiple molecular mechanisms contribute to the recruitment of Asl to centrosomes (related to Figure 2 and Figure 3). (A) Schematic illustration of the regions of DSas-4 and Asl required for their interaction with each other and for the interaction of Asl with Sak/Plk4 [2]. Red boxes indicate the regions of DSas-4 and Asl, respectively, against which the inhibiting antibodies used in our experiments were raised. (B,C) Images show an example of an embryo antibody injection

experiment. The embryo shown here expressed Asl-GFP (*green*) and was injected during mitosis (B) with Texas-Red-labelled anti-DSas-4 antibodies (*red*) in the bottom right corner. The centrosomes close to the injection site are exposed to a high concentration of the antibodies, which binds to the centrosomes; the centrosomes further away show no detectable antibody signal and serve as internal controls. As the centrosomes separate in *S*-phase (C), Asl-GFP is detectable on the new centrosomes in the control area but not near the injection site, where the anti-DSas-4 antibodies decorate the new centrosomes—this is more easily seen in the high magnification images shown in Figure 2A. Scale bar: 10μm. (D,E) Images and graphs show Asl-GFP fluorescence (green) recovery rates following photobleaching of Asl-GFP at old centrosomes, either in a control area or an area near to the injection site following an injection of Texas-Red-labelled antibodies against either DSas-4 (D) or Asl (E); near the injection site, the old centrosomes are decorated with the injected antibodies (red). The initial recovery rates (fluorescence units/sec, during the first 180s after photobleaching) are shown in the graphs to the right of the images. The anti-DSas-4 antibodies (D) do not significantly perturb the turnover of mobile fraction of Asl (n=8 centrosomes from 3 embryos; p=0.58), while the anti-Asl antibodies (E) strongly perturb the turnover of the mobile fraction of Asl (n=8 centrosomes from 3 embryos; p=< 0.0001). Scale bars: 1μm. Error bars represent s.e.m. (F) Graph (with explanatory schematic) showing that old centrioles (that contain Asl—*blue*) and new centrioles (that lack Asl—*orange*) contain similar levels of DSas-4-GFP fluorescence at the time when they first separate and start duplication in early *S*-phase. Later in *S*-phase, the old centrosomes (*blue*) have incorporated additional DSas-4-GFP, indicating that new daughter centrioles have started to assemble, despite the presence of the anti-Asl antibodies; in contrast, the new centrioles (orange) have not incorporated any additional DSas-4, indicating that they are not assembling new daughter centrioles (n=20 centrosomes from 2 embryos for each category). Time relative to centriole separation at t=0 is shown in brackets under the graph. Statistical significance was calculated using a paired two-tailed *t*-test for each comparison. Error bars represent s.e.m.

SUPPLEMENTAL EXPERIMENTAL PROCEDURES

Fly strains and genetics. The Asl-GFP [3] and RFP-PACT [4] constructs used in this study have been described previously. The DSas-4-GFP construct was made by cloning the genetic region of *dsas-4*, from 2kb upstream of the start codon up to the stop codon, into the pUAST-GFPCT vector (Drosophila Genomics Resource Centre, pTWG-1076). The RFP-DSpd-2 construct was generated by cloning the *dspd-2* coding sequence into the pWUbq-RFP(NT) [4] Gateway vector. Transgenic lines were generated at the Department of Genetics, University of Cambridge. For all experiments, Asl-GFP was expressed in an asl^{B46} genetic null mutant background (in which the 5' UTR as well as the first 1775 bp of the *asl* coding region have been deleted — Janina Baumbach, A. W., Z.A.N. and J. W. R., manuscript in preparation), while DSas-4-GFP was expressed in a $sas4^{S2214}$ mutant [5] background.

Microscopy. 1-2h old living embryos were dechorionated by hand on sticky tape, mounted on a thin film of glue (sticky tape dissolved in heptane) on a glass bottom dish, covered with Voltalef oil and imaged immediately at 22^0C. For all imaging experiments shown in this study, with the exception of the super resolultion analysis, embryos were imaged on a Perkin Elmer ERS Spinning Disc confocal system on a Zeiss Axiovert microscope (using a 63x, 1.4NA, oil objective and a charge-coupled device camera: Orca ER, Hamamatsu Photonics). 15-17 confocal sections (0.5µm steps) were collected every 20 seconds. For photobleaching the GFP signal of individual centrosomes, a focused 488nm (when imaging GFP alone) or 440nm laser (when imaging Texas Red or RFP together with GFP) was used. For super resolution analyses (Figure 1 G-I) living embryos were imaged on OMX V3 (GE Healthcare, USA) system (60x, 1.35NA oil objective). 20-23 optical sections were collected to create each image (0.125µm steps).

Image analysis. All images acquired on the Perkin Elmer ERS Spinning Disc confocal system were analysed using Volocity software (Perkin Elmer, USA). To evaluate the changes in Asl-GFP or DSas-4-GFP fluorescence levels during the mitotic cycle, centrosomal fluorescence was calculated as a sum intensity of every centrosomal voxel for each channel (a centrosomal voxel was defined as having an intensity >4.5 standard deviations (SDs) above the mean intensity of all voxels within

the image; this setting was chosen because it selects centrosomal pixels most accurately as judged by eye while allowing consistent thresholding of centrosomal pixels above background fluorescence). Cytoplasmic background fluorescence was then subtracted from centrosomal fluorescence values (background fluorescence within centrosome at timepoint n = mean cytoplasmic fluorescence in timepoint n * number of centrosomal voxels in timepoint n). At each time point centrosomal fluorescence values were corrected for bleaching during sampling using the following method: corrected centrosomal fluorescence at timepoint n = centrosomal fluorescence at timepoint n / (total fluorescence of image at timepoint n/total fluorescence of image at timepoint 1). For DSas-4-GFP, >20 centrosomes were analysed in each of 12 embryos in total (3 embryos (>25 centrosomes each) shown in Figure 1B). For Asl-GFP, 15-25 centrosomes were analysed from each of 10 embryos in total (3 embryos (>15 centrosomes each) shown in Figure 1D).

For comparing Asl-GFP or RFP-PACT levels of centrosomes in *S*-phase (Figure S2B), centrosomal fluorescence was calculated as described above and then local cytoplasmic background fluorescence was subtracted from these values. Fluorescence values of old and young centrosome pairs were compared using a paired *t*-test.

To measure centrosomal Asl-GFP or DSas-4-GFP levels in all antibody-injection experiments (Figures 2C,F; 3B; S3F) or any FRAP experiments (Figures S1F,G; S2D; S3D,E), centrosomal fluorescence was calculated using a different method: we measured the total GFP fluorescence in a boxed region centered around the brightest centrosomal voxel and subtracted the local cytoplasmic background fluorescence. Fluorescence values of inhibited and control centrosomes (Figure 2C,F) were compared using a *t*-test (unpaired). To compare Asl-GFP fluorescence recovery in all antibody-injection experiments (Figure S3D,E) the initial rate of Asl-GFP recovery was calculated by linear regression analysis on fluorescence values measured during the first 180 seconds after photobleaching (10 measurements). A total of 8 centrosomes were analysed from 3 embryos for each group, and recovery rates were compared using a *t*-test (unpaired). To assess DSas-4-GFP recruitment to centrosomes following Asl inhibition, >25 centrosome pairs were analysed from 3 embryos in total. All 3 embryos showed the same qualitative result; in Figure 3B, data from a single embryo (10 Asl-positive and 10 Asl-negative centrosomes) is shown, as the

continuous tracking of Asl-negative centrosomes in the other embryos was more affected by the defective separation and movement of these centrosomes (most likely due to the recruitment of only minimal amounts of PCM in the absence of Asl), in Figure S3F 20 Asl-positive and 20 Asl-negative centrosomes are shown in early (0 sec) and late *S*-phase (140 sec later), from 2 embryos. Fluorescence values were compared using a paired *t*-test.

In all graphs that display data collected from individual embryos (Figure 1B,D; Figure 3B, Figure S2E) the error bars represent s.d., while in all graphs that display data pooled from multiple embryos (Figure 2C,F; Figure S1F,G; S2B,D; Figure S3D,E,F) the error bars represent s.e.m.

All super-resolution images acquired on the OMX V3 system were processed and reconstructed using SoftWorx software. For further analyses, Image J software was used for plotting the distribution of DSas-4-GFP and Asl-GFP within centrosomes. Images were processed as maximum intensity projections; each pixel of the image was sub-divided into 10x10 pixels before the radial intensity profile was calculated from the centre of each centrosome to a 1μm radius; $n_{\text{DSas-4-GFP}} = 8$ centrosomes; $n_{\text{Asl-GFP}} = 24$ centrosomes. Note that in our analysis of DSas-4-GFP the improved resolution allowed us to visualize centriole pairs at virtually all stages of the cell cycle. For ease of presentation, however, we present an image in Figure 1G where one centriole was below the plane of the other centriole, allowing us to obtain a maximum intensity profile through a single centriole; the average fluorescence intensity profile presented in Figure 1H was obtained from 8 such centrioles. This was not necessary for our analysis of Asl-GFP, as the ring of Asl-GFP was only detectable around one centriole (the mother) in each pair. Note that this super-resolution imaging leads to very rapid bleaching of DSas-4-GFP and Asl-GFP, so we could not perform high quality time lapse super-resolution imaging.

In all experiments, cell cycle stage was determined based on the timing of centrosome separation (indicating the start of *S*-phase) and nuclear envelope breakdown (NEB) (indicating the start of mitosis).

With the exception of Figure 1G,I all fluorescent images shown were obtained after 3D rendering of image data in Volocity, and the Volocity 'smooth zoom filter' was applied to all images. Images shown in Figure 1G,I are maximum intensity projections generated in Image J software with no filter applied.

Antibody injections. Anti-DSas-4 [5] or anti-Asl [1] antibodies used for immuno-inhibition experiments were covalently coupled to Texas Red as described previously [6]. The antibodies were injected into embryos at the onset of mitosis. Embryos were imaged immediately following antibody injection using the spinning disc confocal system described above. For FRAP experiments 3-4 old centrosomes were bleached in *S*-phase both on the side of the embryo closest to the injection site (antibody-blocked) and the side furthest form the injection site (control). Data shown was collated from at least 3 embryos for each experiment (see image analysis section above). We have shown previously that the injection of control IgG antibodies does not detectably interfere with the centrosome cycle or with any other aspect of embryo development [6], and have also shown examples where injected anti-centrosomal antibodies that were bound to centrosomes did not detectably perturb centrosome function [1].

Analysis of protein levels in whole embryo lysates. Embryos were dechorionated by bleach and then fixed in methanol containing 2mM EGTA, incubated in methanol at 4^0C for at least 24h, then re-hydrated in PBS+0.1% Triton X-100. 20 embryos were selected manually and homogenized in a total volume of 25µl SDS sample buffer. 10µl of each sample was separated on 3-8% NuPAGE acrylamide gels (Invitrogen) and then transferred to nitrocellulose membranes (0.2µm pore size, BIO-RAD). Membranes were blocked for 1h at room temperature in blocking solution (PBS+4% milk powder+0.1% Tween-20) before incubation with the primary antibody for 1h at room temperature (antibodies were diluted to 1-5ng/ml in blocking solution). Membranes were washed in PBS+0.1% Tween-20 and incubated with horseradish peroxidase-conjugated secondary antibody (GE Healthcare, diluted 3000-fold in blocking solution) for 45 minutes at room temperature. Following washing in PBS+0.1% Tween-20 membranes were incubated with enhanced chemiluminescent substrate for 1 minute (Thermo Scientific, #34095, diluted 2-5 -fold) and exposed to X-ray film.

SUPPLEMENTAL REFERENCES:

S1. Conduit, P.T., Brunk, K., Dobbelaere, J., Dix, C.I., Lucas, E.P., and Raff, J.W. (2010). Centrioles regulate centrosome size by controlling the rate of Cnn incorporation into the PCM. Curr Biol *20*, 2178-2186.

S2. Dzhindzhev, N.S., Yu, Q.D., Weiskopf, K., Tzolovsky, G., Cunha-Ferreira, I., Riparbelli, M., Rodrigues-Martins, A., Bettencourt-Dias, M., Callaini, G., and Glover, D.M. (2010). Asterless is a scaffold for the onset of centriole assembly. Nature *467*, 714-718.

S3. Blachon, S., Gopalakrishnan, J., Omori, Y., Polyanovsky, A., Church, A., Nicastro, D., Malicki, J., and Avidor-Reiss, T. (2008). Drosophila asterless and vertebrate Cep152 Are orthologs essential for centriole duplication. Genetics *180*, 2081-2094.

S4. Lucas, E.P., and Raff, J.W. (2007). Maintaining the proper connection between the centrioles and the pericentriolar matrix requires Drosophila centrosomin. J Cell Biol *178*, 725-732.

S5. Basto, R., Lau, J., Vinogradova, T., Gardiol, A., Woods, C.G., Khodjakov, A., and Raff, J.W. (2006). Flies without centrioles. Cell *125*, 1375-1386.

S6. Gergely, F., Kidd, D., Jeffers, K., Wakefield, J.G., and Raff, J.W. (2000). D-TACC: a novel centrosomal protein required for normal spindle function in the early Drosophila embryo. EMBO J *19*, 241-252.
